# A calmodulin-like protein suppresses RNA silencing and promotes geminivirus infection by degrading SGS3 via the autophagy pathway in *Nicotiana benthamiana*

**DOI:** 10.1371/journal.ppat.1006213

**Published:** 2017-02-17

**Authors:** Fangfang Li, Nan Zhao, Zhenghe Li, Xiongbiao Xu, Yaqin Wang, Xiuling Yang, Shu-Sheng Liu, Aiming Wang, Xueping Zhou

**Affiliations:** 1 State Key Laboratory for Biology of Plant Diseases and Insect Pests, Institute of Plant Protection, Chinese Academy of Agricultural Sciences, Beijing, China; 2 State Key Laboratory of Rice Biology, Institute of Biotechnology, Zhejiang University, Hangzhou, Zhejiang, China; 3 Ministry of Agriculture Key Laboratory of Agricultural Entomology, Institute of Insect Sciences, Zhejiang University, Hangzhou, Zhejiang, China; 4 Southern Crop Protection and Food Research Centre, Agriculture and Agri-Food Canada, London, Ontario, Canada; The Ohio State University, UNITED STATES

## Abstract

A recently characterized calmodulin-like protein is an endogenous RNA silencing suppressor that suppresses sense-RNA induced post-transcriptional gene silencing (S-PTGS) and enhances virus infection, but the mechanism underlying calmodulin-like protein-mediated S-PTGS suppression is obscure. Here, we show that a calmodulin-like protein from *Nicotiana benthamiana* (NbCaM) interacts with Suppressor of Gene Silencing 3 (NbSGS3). Deletion analyses showed that domains essential for the interaction between NbSGS3 and NbCaM are also required for the subcellular localization of NbSGS3 and NbCaM suppressor activity. Overexpression of NbCaM reduced the number of NbSGS3-associated granules by degrading NbSGS3 protein accumulation in the cytoplasm. This NbCaM-mediated NbSGS3 degradation was sensitive to the autophagy inhibitors 3-methyladenine and E64d, and was compromised when key autophagy genes of the phosphatidylinositol 3-kinase (PI3K) complex were knocked down. Meanwhile, silencing of key autophagy genes within the PI3K complex inhibited geminivirus infection. Taken together these data suggest that NbCaM acts as a suppressor of RNA silencing by degrading NbSGS3 through the autophagy pathway.

## Author summary

Post-transcriptional gene silencing (PTGS) is an elaborately regulated process for defense against virus infection in plants. To achieve effective infection, a betasatellite molecule associated with geminivirus induced high levels of an endogenous RNA silencing suppressor, calmodulin-like protein (CaM), to counter host defenses. However, although CaM is one of the first identified cellular suppressors of RNA silencing, the mechanism of PTGS suppression is still poorly understood. This study demonstrates that CaM interacts with and degrades Suppressor of Gene Silencing 3 (SGS3) in *Nicotiana benthamiana*. We found that domains essential for the interaction between NbSGS3 and NbCaM are also required for the subcellular localization of NbSGS3 and for NbCaM suppressor activity. Moreover, NbCaM mediated NbSGS3 protein degradation can be blocked using the autophagy inhibitors 3-methyladenine and E64d, and by knock-down of key autophagy-related genes within the phosphatidylinositol 3-kinase (PI3K) complex. Silencing of the PI3K complex also inhibited geminivirus infection, which is consistent with autophagy playing an important role in RNA silencing suppression pathway and geminivirus infection.

## Introduction

Post transcriptional gene silencing (PTGS) is an important RNA interference (RNAi)-based defense mechanism against foreign nucleic acid invasion and is involved in silencing a wide range of endogenous genes in plants. PTGS is triggered by double-stranded RNAs (dsRNAs), which are cleaved into 21- to 24- nucleotide (nt) small interfering RNA (siRNA) duplexes by Dicer-like (DCL) endoribonucleases. Subsequently, the siRNAs are loaded into an RNA-induced silencing complex (RISC), which contains an RNaseH-like Argonaute (AGO) enzyme, and one strand of the siRNA duplex is used to guide AGO to cleave homologous RNAs for degradation [1, 2].

In plants, overexpressed transgene transcripts, viral RNAs or their cleavage products can serve as the substrates for RNA-dependent RNA polymerases (e.g. RDR6) for conversion of single-stranded RNAs (ssRNAs) to dsRNAs, which further produce secondary siRNA molecules through DCL cleavage [3]. Therefore, RDR6 plays a key role in the sense RNA-induced PTGS (S-PTGS) pathway, the synthesis of trans-acting small-interfering RNA (ta-siRNAs), and anti-viral silencing pathways [4–6]. Recently, many reports have shown that RDR6-deficient plants (e.g. *Nicotiana benthamiana*) are more susceptible to infection by some positive-sense single-stranded RNA viruses, viroids and DNA viruses [7–10], strongly supporting the role of RDR6 in the host antiviral response. In these processes, a plant-specific RNA binding protein, Suppressor of Gene Silencing 3 (SGS3), functions together with RDR6 as a chaperone protein [4–6]. *Arabidopsis* SGS3 (AtSGS3) contains a zinc finger (ZF), rice gene X and SGS3 (XS), and coiled-coil (CC) domain. Among these, the XS and CC domains are involved in RNA binding and homodimer formation, respectively, and both are required for normal AtSGS3 localization and function in the synthesis of ta-siRNAs in plants [11–13]. Previous studies have suggested that AtSGS3 binds and stabilizes RNA templates during initiation of *Arabidopsis* RDR6 (AtRDR6)-mediated dsRNA synthesis [14], and AtSGS3 and AtRDR6 co-localize in certain cytoplasmic granules called SGS3/RDR6-bodies [13]. However, whether SGS3 from *N*. *benthamiana* plays a similar chaperone role with NbRDR6 is still obscure.

PTGS is an elaborately regulated process targeted against viral infection. However, most plant viruses have evolved viral suppressors of RNA silencing (VSRs) to counteract host antiviral silencing activity. Various VSRs have been identified in almost all plant virus genera, but they exhibit no obvious sequence similarities and interact with RNA-silencing pathways in multiple ways [15]. Recent reports show that several components of the *Arabidopsis* cytoplasmic exoribonuclease complex participated in mRNA quality control and mRNA processing, including FIERY1, XRN2, XRN3, XRN4, EIN5 and SKI2, which can also function as repressors of PTGS [16–18]. Moreover, impairing nonsense-mediated decay, deadenylation or exosome activity enhances S-PTGS in *Arabidopsis*, which requires the host RDR6 and SGS3 proteins for conversion of ssRNAs into dsRNAs to trigger PTGS [19]. Those endogenous RNA suppressors derived from mRNA decay pathways competed for SGS3/RDR6 RNA substrates to repress RNA silencing, suggesting the crucial role of SGS3/RDR6 in the endogenous RNA silencing pathway.

A calmodulin-like protein from *Nicotiana tabacum* (NtCaM) has been identified as an endogenous RNA silencing suppressor which interacts with the helper component-proteinase (HC-Pro) of a potyvirus [20]. However, follow-up work showed that NtCaM interacts with and directs degradation of several dsRNA binding VSRs likely through the autophagy-like protein degradation pathway, revealing a contradictory function for NtCaM in antiviral defense [21]. Nevertheless, a growing body of evidence published recently by different laboratories supports a role for the calmodulin-like protein as an S-PTGS suppressor [10, 22–24]. In the case of geminivirus infections, calmodulin-like protein from *N*. *benthamiana* (NbCaM) was up-regulated by *Tomato yellow leaf curl China betasatellite* (TYLCCNB)-encoded βC1 upon virus infection. Up-regulation of NbCaM by βC1 suppressed RNA silencing by repressing expression of RDR6 to promote viral infection [10]. Moreover, overexpression of *Arabidopsis* calmodulin-like protein 39 (AtCaM39) leads to increased susceptibility to infection by *Tomato golden mosaic virus* (TGMV) [22]. These studies indicate that calmodulin-like proteins are hijacked by plant viruses (at least geminiviruses, if not all) to counterattack the host defense response. However, the precise mechanism of calmodulin-like protein-mediated S-PTGS suppression is yet to be understood.

Autophagy is thought to be a nonspecific, bulk degradation process by which eukaryotic cells recycle intracellular components, such as protein aggregates and organelles [25]. There are at least three types of autophagy: macroautophagy, microautophagy and chaperone-mediated autophagy [26]. Macroautophagy (hereafter referred to as autophagy) is the major type of autophagy, and it occurs when cytoplasmic constituents are engulfed by double-membrane vesicles termed autophagosome and subsequently delivered to the vacuoles for breakdown and turnover in plants [27]. Autophagy is evolutionarily conserved from yeast to plants, and most of the essential components have been identified and characterized in plants through comparison to their homologs in yeast [26, 28–30]. Among these autophagy-related genes (*ATGs*), Beclin1 forms a complex with PI3K/VPS34, the class III phosphatidylinositol 3-kinase, as a first step in the initiation of autophagy, recruits other proteins to the complex and is required for autophagosome formation [26, 31].

Autophagy has also been shown to be important for anti-viral defense. In *Drosophila*, ATGs protect against *Vesicular stomatitis virus* (VSV) infection, and disruption of ATG5, ATG8, and ATG18 is associated with increased VSV RNA replication resulting in increased animal lethality [32]. Autophagy has also been reported to participate in antiviral defense in mammalian systems. For example, *ATG5* is essential to protect mice against lethal infection of the mouse central nervous system by *Sindbis virus* [33]. Not surprisingly, viruses have developed strategies to subvert or use autophagy for their own benefit. For example, autophagy proteins are proviral factors that favor initiation of *Hepatitis C virus* infection and are required for translation of incoming viral RNA [34, 35]. In plants, deficiency in *ATGs* compromises plant vitality and disease resistance [29, 36, 37]. For example, *N*-gene mediated resistance against *Tobacco mosaic virus* (TMV) is dependent on autophagy genes, and plants deficient in the autophagy genes, *Beclin1*, *PI3K*/*VPS34*, *ATG3*, and *ATG7*, exhibit an unrestricted hypersensitive response (HR) in response to pathogen infection [29]. However, *Arabidopsis* mutant *atg2-2* and several other *ATG* mutants, including *atg5*, *atg7* and *atg10*, exhibit enhanced resistance to powdery mildew and dramatic mildew-induced cell death [38], providing new insights into the role of autophagy in disease resistance and cell death. A recent report showed that autophagy is possibly involved in the RNA silencing suppressor activity of P0, as P0-mediated degradation of AGO1 can be blocked by autophagy inhibitors [39]. Therefore, there is a question of whether autophagy is involved in the suppressor activity of NbCaM or in geminivirus infection.

In this study, we show that NbCaM interacts with SGS3 from *N*. *benthamiana* (NbSGS3), but not with NbRDR6. Furthermore, we found that NbCaM induces degradation of NbSGS3 by interacting with ATG factors, and silencing of *ATG* genes inhibits NbCaM-mediated NbSGS3 degradation and promotes resistance to infection by the geminivirus *Tomato yellow leaf curl China virus* (TYLCCNV) and its betasatellite (TYLCCNB). Together with previous results, these findings suggest that the endogenous RNA silencing suppressor NbCaM regulates RNA silencing and promotes geminivirus infection by repressing *NbRDR6* expression and promoting degradation of NbSGS3, most likely via the autophagy pathway.

## Results

### NbCaM interacts with NbSGS3, but not with NbRDR6

**Fig 1:**
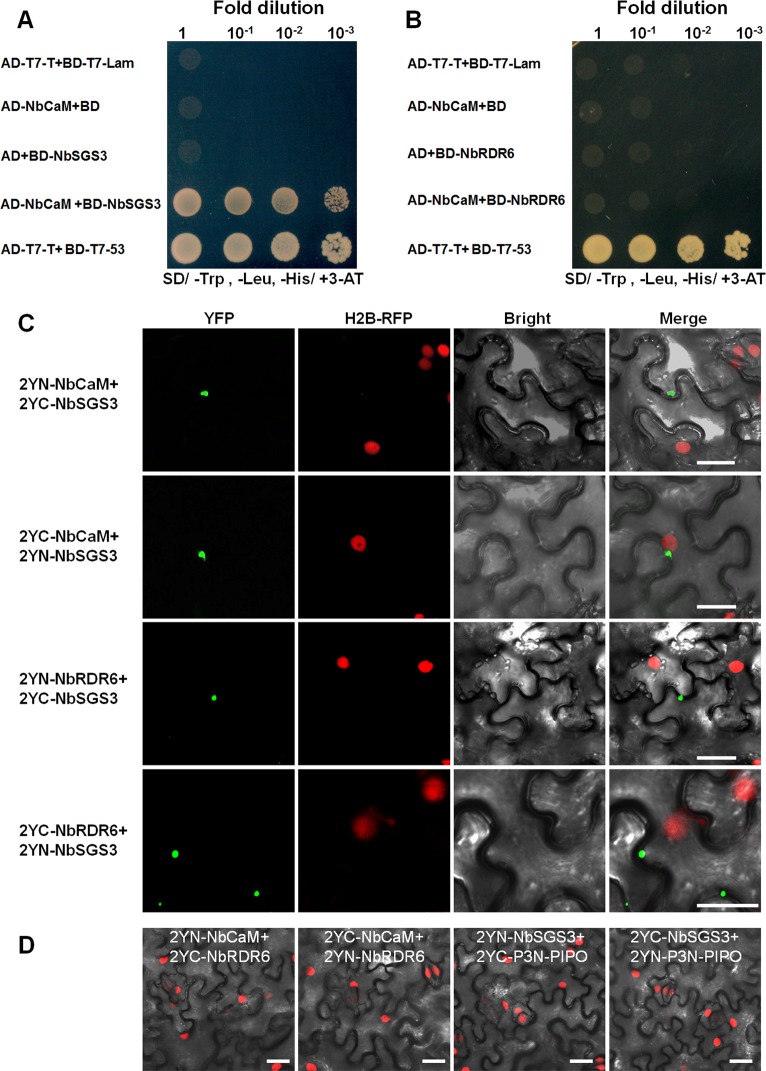
Interactions between NbCaM, NbSGS3 and NbRDR6. **(A** and **B)** Yeast two-hybrid assays using NbCaM and NbSGS3 (A) or NbCaM and NbRDR6 (B). Serial 10-fold dilutions of yeast cells were made as indicated. Cells co-transformed with AD-T7-T+BD-T7-53 served as a positive control, and cells co-transformed with AD-T7-T+BD-T7-Lam, or with empty vectors pGBKT7 (BD) and pGADT7 (AD) served as negative controls. BD, GAL4 DNA binding domain; AD, GAL4 activation domain. **(C** and **D)** BiFC assays using NbCaM, NbSGS3 and NbRDR6 in H2B-RFP transgenic *N*. *benthamiana* leaves at 48 hours post infiltration. YFP fluorescence (green) was observed as a consequence of complementation of NbCaM and NbSGS3, or NbSGS3 and NbRDR6 tagged with 2YN and 2YC. Nuclei of tobacco leaf epidermal cells are indicated by expression of the H2B-RFP transgene (red). No YFP fluorescence was found between pairwise expression NbCaM and NbRDR6, or NbSGS3 and P3N-PIPO (**D**). Single channels from the merged image shown in (**D**) are shown in S2 Fig. Bars = 25 μm.

NbCaM suppresses sense RNA-induced PTGS and enhances geminivirus infection in *N*. *benthamiana*, similar to results observed when expression of *AtSGS3* and *AtRDR6* in *Arabidopsis* is reduced [4–6]. To explore the molecular mechanism of NbCaM-mediated suppression of RNA silencing and augmentation of geminivirus infection, potential interactions among NbCaM, NbSGS3 and NbRDR6 were analyzed initially using yeast two-hybrid (Y2H) assays. An expressed sequence tag (EST) for the *NbSGS3* sequence was identified by aligning tobacco *NtSGS3* and tomato *SlSGS3* sequences (obtained from the GenBank database). Primers were designed to amplify the full-length coding sequence and the full-length gene encoding NbSGS3 was cloned from *N*. *benthamiana*. Sequence analysis revealed that the *NbSGS3* open reading frame (ORF) contains 1908 nucleotides (nt) (GenBank accession number: KJ190939). Co-transformants of NbCaM cloned as a fusion with the GAL4 activation domain (AD-NbCaM) and NbSGS3 as a fusion with the GAL4 DNA binding domain (BD-NbSGS3) were plated on different selective media to detect activation of the reporter genes, *HIS3* and *ADE2*. Yeast transformants containing AD-NbCaM and BD-NbSGS3 were able to grow on SD/Leu-Trp-His selection plates with 5 mM 3-amino-1,2,4-triazole (3-AT), whereas yeast transformants carrying AD-NbCaM with empty vector (AD-NbCaM + BD) or BD-NbSGS3 with empty vector (BD-NbSGS3 + BD) were unable to proliferate (**Fig 1A**). Furthermore, yeast transformants containing AD-NbCaM and BD-NbRDR6 showed no interaction between the proteins tested (**Fig 1B**). In addition, yeast transformants containing AD-NbRDR6 and BD-NbSGS3 or AD-NbSGS3 and BD-NbSGS3 also grew on the selection plates (**S1A and S1B Fig**), consistent with an interaction between SGS3 and RDR6 and self-interaction of SGS3, which was observed for AtSGS3 and AtRDR6 [12, 13]. Expression of NbCaM, NbSGS3 and NbRDR6 proteins was verified by Western blot (**S1C–S1F Fig**).

The interaction between NbCaM, NbSGS3 and NbRDR6 was further investigated by bimolecular fluorescence complementation (BiFC) in leaves from transgenic *N*. *benthamiana* plants which expressed H2B-RFP as a nuclear marker. In this assay, NbCaM, NbSGS3 and NbRDR6 were fused to the N (2YN) and C-terminal (2YC) fragments of yellow fluorescent protein (YFP), generating the constructs 2YN-NbCaM, 2YC-NbCaM, 2YN-NbSGS3, 2YC-NbSGS3, 2YN-NbRDR6 and 2YC-NbRDR6. Pairwise expression of 2YN-NbCaM and 2YC-NbSGS3, 2YC-NbCaM and 2YN-NbSGS3, 2YN-NbRDR6 and 2YC-NbSGS3, and 2YC-NbRDR6 and 2YN-NbSGS3 by agroinfiltration resulted in a clear YFP fluorescence signal in the cytoplasm of agroinfiltrated cells at 48 hours post infiltration (hpi) (**Fig 1C**). While no YFP fluorescence was observed when 2YN-NbCaM and 2YC-NbRDR6 or 2YC-NbCaM and 2YN-NbRDR6 were co-expressed together (**Fig 1D and S2 Fig**). The movement protein P3N-PIPO from *Turnip mosaic virus* (TuMV) [40] was used as a negative control, we found there was no YFP fluorescence in the pairwise expression of 2YN-NbSGS3 and 2YC-P3N-PIPO or 2YC-NbSGS3 and 2YN-P3N-PIPO (**Fig 1D and S2 Fig**). These results demonstrate that NbCaM specifically interacts with NbSGS3 in both yeast and plant cells.

### Sequence analysis, expression pattern and subcellular localization of NbSGS3

**Fig 2:**
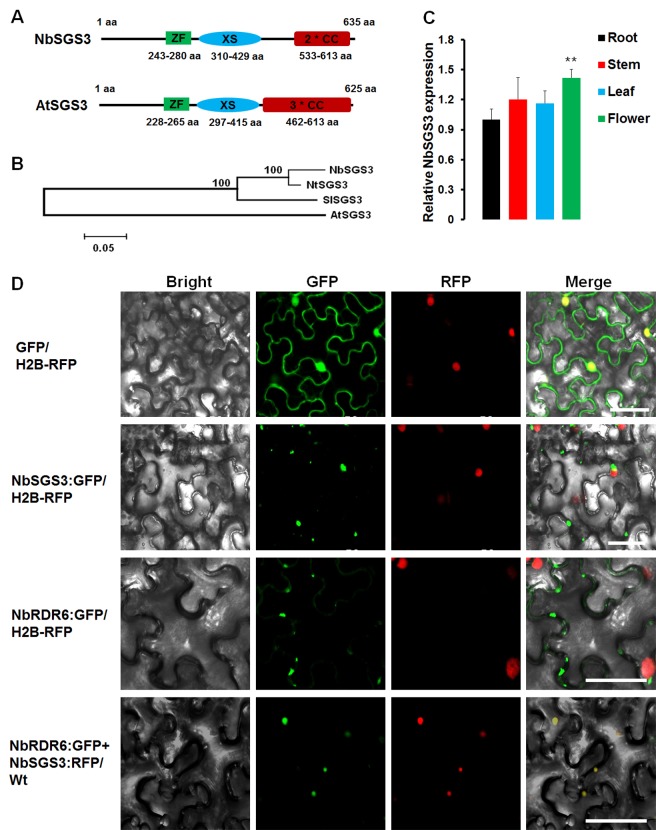
Sequence analysis, expression pattern and subcellular localization of NbSGS3. **(A)** The linear diagram represents the domain structure of AtSGS3 and NbSGS3, ZF: Zinc finger, XS: Rice protein X and SGS3, CC: Coiled-coil. **(B)** Phylogenetic tree showing the evolutionary relationships between NbSGS3, NtSGS3, SlSGS3 and AtSGS3 proteins. Amino acid sequence alignment was performed in ClustalX2 and tree construction was conducted in MEGA6 using the Neighbour Joining method. The scale bar represents the number of changes per site. **(C)** RT-qPCR analysis of *NbSGS3* expression levels in different tissues of *N*. *benthamiana*. Expression was normalized to *NbGAPDH* levels, which serves as an internal standard. The relative mRNA level of *NbSGS3* in root was arbitrarily set to 1. R: Root, S: Stem, L: Leaf, F: Flower. Student’s *t* test was performed to compare differences between root and stem, leaf or flower tissues. Each mean value was derived from three independent experiments (n = 9). Values represent the mean ± standard deviation (SD). Double asterisks indicate a highly significant difference (p<0.01) between root and flower tissues. **(D)** Micrographs showing cells from leaves of H2B-RFP transgenic *N*. *benthamiana* expressing GFP, NbSGS3:GFP or NbRDR6:GFP (panels in top three rows), or NbRDR6:GFP and NbSGS3:RFP in Wt *N*. *benthamiana* (bottom row). Bars = 50 μm.

In *Arabidopsis thaliana*, efficient RNA silencing requires RDR6 and its double-stranded RNA (dsRNA)-binding partner, SGS3 to amplify secondary siRNAs which allow plants to mount an effective defense response against transgene-induced aberrant RNAs or virus infection [4–6]. To better understand the role of NbCaM in the S-PTGS pathway and the potential effect of NbCaM on the function of NbSGS3, the sequence and biological features of NbSGS3 were analyzed. NbSGS3 cDNA encodes a 635-amino acid (aa) protein, with a structure similar to AtSGS3 and contains a zinc finger domain (ZF), a rice gene X and SGS3 domain (XS), and two coiled-coil domains (2*CC) (**Fig 2A**). A phylogenetic tree was constructed to compare the evolutionary relationships among SGS3 orthologs in tobacco (NtSGS3), tomato (SlSGS3) and *Arabidopsis* (AtSGS3) (**Fig 2B**). NbSGS3 is clustered with NtSGS3 and SlSGS3 and is distant from AtSGS3, sharing 94%, 82% and 51% aa identity with NtSGS3, SlSGS3 and AtSGS3, respectively.

To determine the expression pattern of *NbSGS3*, reverse transcription real-time quantitative PCR (RT-qPCR) was performed using total RNA isolated from different *N*. *benthamiana* tissues as template. *NbSGS3* expression levels were very similar among different tissues, with the exception that the expression level in flower was higher than that detected in root tissues (**Fig 2C**, p<0.01). To examine the subcellular localization of NbSGS3, a green (GFP) or red (RFP) fluorescent protein reporter was fused to its C-terminus (NbSGS3:GFP or NbSGS3:RFP) under control of the *Cauliflower mosaic virus* (CaMV) 35S promoter. GFP or NbSGS3:GFP was transiently expressed in leaves of transgenic H2B-RFP *N*. *benthamiana* plants, and GFP fluorescence was examined in agroinfiltrated transgenic leaves at 48 hpi by confocal microscopy. Fluorescence in plants expressing GFP alone was observed in both the cytoplasm and nucleus, whereas fluorescence from NbSGS3:GFP was localized to granular-like structures in the cytoplasm (**Fig 2D**). These granular-like structures were also observed in *N*. *benthamiana* protoplasts (**S3A Fig**). Similar granular-like structures were also observed when a YFP was fused to the N terminus of NbSGS3 (YFP:NbSGS3, **S3B Fig**). A previous study reported that AtSGS3 localized to cytoplasmic granules, termed SGS3/RDR6-bodies [13]. To check whether NbSGS3 also localizes to SGS3/RDR6-bodies, NbRDR6:GFP and NbSGS3:RFP were co-expressed in wild type (Wt) *N*. *benthamiana*. As shown in **Fig 2D**, NbRDR6 alone formed irregular granules along the edge of the cell (the third line), but NbRDR6:GFP co-localized with NbSGS3:RFP to the cytoplasmic granules (the fourth line). In addition, we also examined whether NbSGS3 granules are related to cellular organelles, including chloroplasts, mitochondria, golgi bodies or peroxisomes, but no co-localization was found (**S3A Fig** and **S4 Fig**).

### Functional characterization of conserved domains in NbCaM and NbSGS3

**Fig 3:**
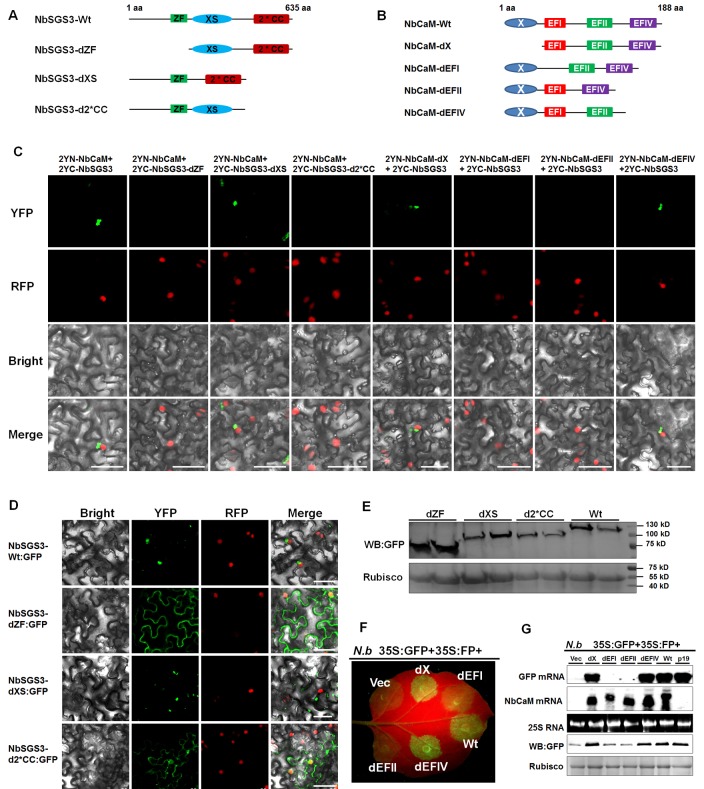
Function of conserved domains in NbCaM and NbSGS3. **(A)** Schematic representation of wild type NbSGS3 and its deletion derivatives. ZF: zinc finger domain; XS: rice gene X and SGS3 domain; 2*CC: two coiled-coil domains. **(B)** Schematic representation of wild type NbCaM and its deletion derivatives. X: an unknown domain; EFI: first Ca^2+^ binding domain; EFII: second Ca^2+^ binding domain; EFIV: fourth Ca^2+^ binding domain. **(C)** BiFC analysis of NbCaM and NbSGS3. Deletion mutants fused to the N- or C-terminal part of YFP are indicated. Bars represent 50 μm. **(D)** Subcellular localization of GFP fused to wild type NbSGS3 (NbSGS3-Wt:GFP), ZF (NbSGS3-dZF:GFP), XS (NbSGS3-dXS:GFP), or 2*CC (NbSGS3-d2*CC:GFP) deletion mutant. Bars represent 50 μm. **(E)** Western blot analysis of total protein extracts of samples shown in (D). Ponceau staining of Rubisco large subunit is used as a loading control. **(F)** GFP fluorescence in leaves of *N*. *benthamiana* plants co-infiltrated with *Agrobacterium* cultures containing 35S:GFP+35S:FP and either empty control (Vec), the four deletion mutants of NbCaM or wild type NbCaM as indicated. Infiltrated leaves were photographed at 5 dpi under UV light. **(G)** Analyses of RNA and protein levels in the agroinfiltrated leaf samples shown in (F). TBSV p19 was used as a positive control for silencing suppression.

To map the protein domains required for the interaction between NbCaM and NbSGS3, three deletion mutants for NbSGS3 and four deletion mutants for NbCaM were constructed (**Fig 3A and 3B**). Mutants NbCaM-dX, NbCaM-dEFI, NbCaM-dEFII and NbCaM-dEFIV lacking the N-terminal 50 aa of an unknown domain, or the first, second and fourth Ca^2+^ binding EF-hand domain, respectively, were cloned into the plant BiFC vector 2YN [41]. Mutants NbSGS3-dZF, NbSGS3-dXS and NbSGS3-d2*CC, which lack the zinc finger, rice gene X and SGS3 domain, and two coiled-coil domains, respectively, were generated and cloned into the plant BiFC vector 2YC [41]. BiFC assays were performed using 2YN- and 2YC-tagged mutant proteins in transgenic H2B-RFP *N*. *benthamiana* plants. NbCaM deletion mutants NbCaM-dEFI and NbCaM-dEFII were unable to interact with NbSGS3, and NbSGS3 deletion mutants NbSGS3-dZF and NbSGS3-d2*CC failed to interact with NbCaM (**Fig 3C**). These results suggest that the EFI and EFII domains of NbCaM and the ZF and CC domains of NbSGS3 are essential for the interaction between NbSGS3 and NbCaM.

Localization of SGS3 to SGS3/RDR6-bodies is one of its basic features [13]. To investigate whether the domains essential for the interaction between NbCaM and NbSGS3 are also important for localization of NbSGS3, three deletion mutants of NbSGS3 were fused with GFP at their C-termini. Typical localization patterns of the deletion mutants and wild type NbSGS3:GFP (NbSGS3-Wt:GFP) are shown in **Fig 3D**. Expression of these proteins was verified by Western blot analyses (**Fig 3E**). In contrast to the granule localization of NbSGS3-Wt:GFP and the NbSGS3-dXS:GFP mutant, the NbSGS3-dZF:GFP or NbSGS3-d2*CC:GFP mutant exhibited no obvious granule localization (**Fig 3D**). These results suggest that the ZF and the 2* CC domains are important for localization of NbSGS3 to the granules.

We next tested whether domains essential for the interaction between NbCaM and NbSGS3 are also required for the PTGS suppressor activity of NbCaM. *N*. *benthamiana* leaves were co-infiltrated with agrobacteria harboring either an empty vector (Vec), 35S:NbCaM-dX (dX), 35S:NbCaM-dEFI (dEFI), 35S:NbCaM-dEFII (dEFII), 35S:NbCaM-dEFIV (dEFIV) or 35S:NbCaM (wild type NbCaM, Wt), together with 35S:GFP plus 35S:FP to trigger PTGS. Weak GFP fluorescence was observed in tissues co-infiltrated with empty vector, 35S:NbCaM-dEFI or 35S:NbCaM-dEFII together with 35S:GFP +35S:FP at 5 dpi. In contrast, strong fluorescence was evident in infiltrated patches where 35S:GFP +35S:FP were co-expressed with 35S:NbCaM-dX, 35S:NbCaM-dEFIV or 35S:NbCaM (**Fig 3F**). GFP fluorescence in infiltrated leaf patches was confirmed by the presence of GFP mRNA and protein, and expression of Wt or mutant forms of NbCaM was validated by the presence of NbCaM mRNA (**Fig 3G**). These results demonstrate that domains within NbCaM that are essential for the interaction with NbSGS3 are also indispensable for NbCaM suppressor activity.

### NbCaM induces a reduction in the number of NbSGS3 granules and mediates NbSGS3 protein degradation

**Fig 4:**
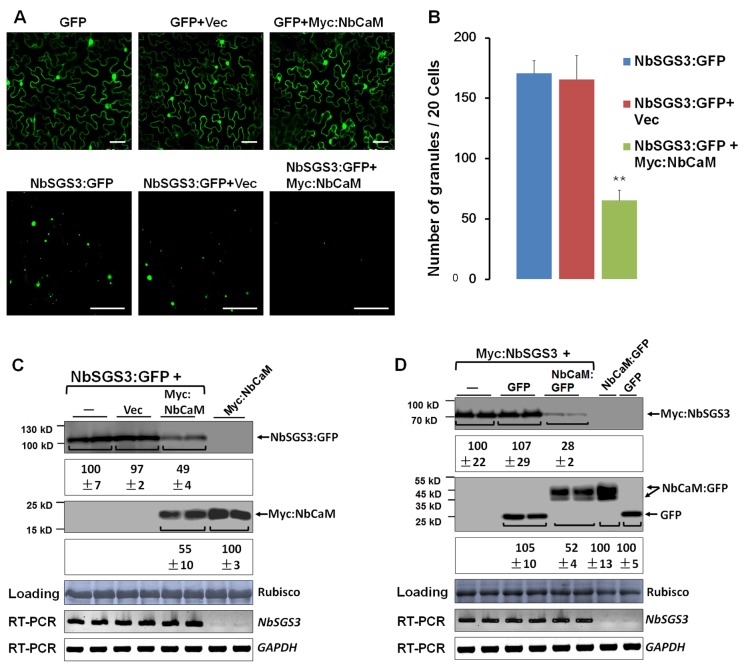
NbCaM reduces the number of NbSGS3 granules and mediates NbSGS3 protein degradation. **(A)** Micrographs showing cells expressing GFP alone, or in conjunction with an empty vector (Vec) or Myc:NbCaM (upper panel), and expressing NbSGS3:GFP alone, or in conjunction with Vec or Myc:NbCaM (lower panel). Infiltrated *N*. *benthamiana* leaves were examined at 48 hpi. Bars represent 50 μm. **(B)** The graph illustrates the average number of NbSGS3:GFP granules per 20 cells. Infiltration experiments were performed three independent times and 20 cells were examined in each experiment (a total of sixty cells). Values presented represent the mean ± standard deviation (SD). Double asterisks indicate a highly significant difference (p<0.01) between plant cells expressing NbSGS3:GFP alone and co-expressing NbSGS3:GFP with Myc:NbCaM (Student’s *t* test). **(C)** Western blot of total protein extracts from (B) detected with GFP (first row) or Myc (third row) antibody. Protein assays were done at least three independent times, and protein bands from two representative experiments were quantified using Image J software (second row and fourth row). The amount of protein detected in samples expressing of NbSGS3:GFP or Myc:NbCaM alone was set at 100 and values under the blots represent the average level of protein ± standard deviation (SD). Coomassie brilliant blue staining of Rubisco large subunit was used as a loading control. RT-PCR was used for analysis of *NbSGS3* transcripts. *NbGAPDH* served as an internal standard. **(D)** Western blot analysis of total protein extracts from leaves infiltrated with Myc:NbSGS3 alone, or together with GFP or NbCaM:GFP. Myc (first row) or GFP (second row) antibody was used. Along with a band consistent with the expected size for NbCaM:GFP (48 kDa), a smaller sized protein band was also detected. This could represent different modified forms of this protein or protein degradation by protease. Protein assays were performed from at least three independent experiments, and protein bands from two representatives were quantified using Image J software (second row and fourth row). The amount of protein detected in samples expressing Myc:NbSGS3, GFP, or NbCaM:GFP alone was set at 100 and values represent the average level of protein ± standard deviation (SD).

It has been reported that the correct localization of SGS3 is important for its biological function [13]. To determine whether NbCaM affects the localization pattern of NbSGS3, GFP and NbSGS3:GFP fusion proteins were expressed alone or co-expressed with either empty vector (Vec) or Myc:NbCaM in *N*. *benthamiana* leaves. The distribution of GFP was very similar between tissue expressing GFP alone, or when co-expressing empty vector or Myc:NbCaM (**Fig 4A**). The NbSGS3:GFP fusion protein formed granules in the cytoplasm when it was expressed alone or together with empty vector. However, when NbSGS3:GFP was co-expressed with Myc:NbCaM, the number of NbSGS3:GFP granules was greatly decreased as compared to plants expressing NbSGS3:GFP alone or in conjunction with empty vector (**Fig 4A and 4B**). To further determine whether the weak fluorescence of NbSGS3:GFP in plants co-expressing Myc:NbCaM was due to decreased NbSGS3:GFP levels, Western blot analysis was performed to determine the accumulation of NbSGS3:GFP. As expected, levels of NbSGS3 decreased ~2-fold when co-expressed with Myc:NbCaM as compared to empty vector (**Fig 4C**). To exclude the possible influence of the tag, Myc:NbSGS3 was also expressed alone, or co-expressed with GFP or NbCaM:GFP. Protein and mRNA levels of Myc:NbSGS3 remained constant when expressed alone or together with GFP. In samples overexpressing NbCaM:GFP, no obvious changes in Myc:NbSGS3 mRNA were observed, but the amount of NbSGS3 protein was largely reduced (**Fig 4D**). It is worth mentioning that the NbCaM protein level was also reduced ~2-fold when co-expressed with NbSGS3 as compared to NbCaM alone (**Fig 4C and 4D**). These results suggest that overexpression of NbCaM leads to reduced NbSGS3 protein accumulation, and that both may be targeted for degradation after they form a complex.

### NbCaM and NbSGS3 act synergistically to induce autophagic activity and NbSGS3 degradation mediated by NbCaM is blocked by autophagy inhibitors

**Fig 5:**
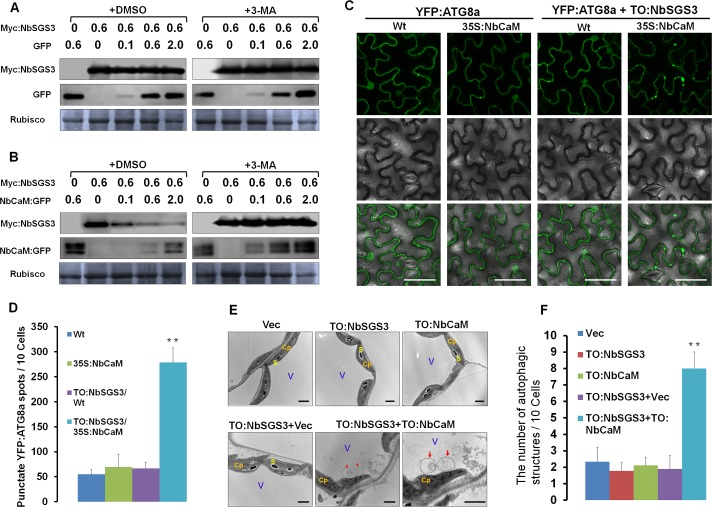
NbCaM-mediated degradation of NbSGS3 is blocked by inhibitors of autophagy, and co-expression of NbCaM and NbSGS3 induces autophagic activity. **(A, B)** Effect of the autophagy inhibitor 3-MA on the accumulation of Myc:NbSGS3 when co-expressed with different concentrations of GFP (A) or NbCaM:GFP (B) at 2 dpi. Samples were analyzed by Western blot using Myc or GFP antibody. The OD_600_ of *Agrobacterium* culture mixtures containing a plasmid capable of expressing Myc:NbSGS3 alone, or together with GFP or NbCaM:GFP are indicated. DMSO or 3-MA (10 mM) was infiltrated into leaves 32 h after being agroinfiltrated with Myc:NbSGS3 along with GFP or NbCaM:GFP constructs. Samples were harvested from 16 h later. Coomassie brilliant blue staining of Rubisco large subunit was used as a loading control. The assays were performed from at least three independent experiments, and one representative result is shown. **(C)** Micrographs showing cells expressing YFP:NbATG8a alone, or together with NbSGS3 (TO:NbSGS3) on wild type (Wt) or 35S:NbCaM transgenic (35S:NbCaM) *N*. *benthamiana* plants. Infiltrated *N*. *benthamiana* leaves were examined at 48 hpi. Bars represent 50 μm. **(D)** The average number of punctate YFP fluorescent structures per 10 cells shown in (C). Independent infiltration experiments were performed three times and 10 cells examined in each experiment to give a total of thirty cells. Values represent the mean ± standard deviation (SD). Double asterisks indicate a highly significant difference (p<0.01) between Vec and NbSGS3 + NbCaM-infiltrated leaves (Student’s *t* test). **(E)** Representative TEM images of *N*. *benthamiana* leaves with the transient overexpression of empty vector (Vec), NbSGS3 (TO:NbSGS3), NbCaM (TO:NbCaM), TO:NbSGS3+Vec or TO:NbSGS3+TO:NbCaM. Obvious autophagic structures (red arrows) were observed in TO:NbSGS3+To:NbCaM infiltrated leaves in the central vacuole of mesophyll cells at 2 dpi. Cp: chloroplast, S: starch, V: vacuole. Bars = 2 μm. (**F**) The number of typical double-membrane autophagosome in leaves infiltrated with TO:NbSGS3, TO:NbCaM, TO:NbSGS3+Vec or TO:NbSGS3+TO:NbCaM, which was normalized to those found in leaves infiltrated with empty vector. Infiltration and TEM observation experiments were repeated twice and approximately 60 cells in total were used to quantify autophagic structures in each treatment. Values represent the mean number of autophagosomes ± standard deviation (SD). Student’s *t* test was performed to compare differences between Vec and NbSGS3, NbCaM, NbSGS3 +Vec or NbSGS3 +NbCaM-infiltrated leaves and double asterisks indicate a highly significant difference (p<0.01).

A recent study showed that NtCaM is sensitive to 3-methyladenine (3-MA) and E64d, inhibitors of the autophagy pathway, and NtCaM seems to mediate degradation of VSRs [21]. To determine whether NbCaM-mediated degradation of NbSGS3 protein occurs via autophagy or other protein degradation systems, the sensitivity of NbCaM or NbSGS3 to 3-MA and E64d, two chemical inhibitors of autophagy, was tested. *N*. *benthamiana* leaves were agroinfiltrated with GFP, NbCaM:GFP or NbSGS3:GFP followed by infiltration of DMSO (control), 3-MA (10 mM) or E64d (100 uM) after 32 hours. Samples were collected from leaves after an additional 16 h incubation. No obvious changes in GFP, NbCaM:GFP and NbSGS3:GFP protein levels were observed in DMSO, 3-MA or E64d treated samples (**S5 Fig**). These results suggest that inhibition of autophagy did not affect accumulation of NbCaM or NbSGS3 when expressed alone. Similarly, the 26S proteasome inhibitor MG132 did not have an observable impact on the accumulation of NbCaM or NbSGS3 (**S6 Fig**). We next co-expressed Myc:NbSGS3 with GFP or Myc:NbSGS3 with NbCaM:GFP in plants treated with DMSO or 3-MA using different concentrations of *Agrobacterium tumefaciens* cultures carrying GFP or NbCaM:GFP and assessed protein levels by Western blot. Levels of GFP and Myc:NbSGS3 protein did not appear to change when co-expressed or when expressed in the presence of DMSO or 3-MA (**Fig 5A**). However, both Myc:NbSGS3 and NbCaM:GFP protein accumulated to higher levels in 3-MA treated plants as compared to DMSO treated plants (**Fig 5B**). The E64d had a similar role with 3-MA on the accumulation of NbSGS3:GFP when it was co-expressed with Myc:NbCaM (**S7 Fig**). These results suggest that NbCaM and NbSGS3 are most likely degraded by autophagy in plant cells. To confirm this assumption, we used YFP-tagged *N*. *benthamiana* ATG8a (YFP-ATG8a) as an autophagosome marker to monitor autophagy [36, 42–45]. In Wt or 35S:NbCaM transgenic *N*. *benthamiana* plants, we observed a low number of punctate YFP fluorescent structures (**Fig 5C**). However, when NbSGS3 was transiently over-expressed in 35S:NbCaM transgenic *N*. *benthamiana* plants via infiltration with TO:NbSGS3, there was a 3 to 4-fold increase in the punctate fluorescent structures, likely representing pre-autophagosome or autophagosome structures (**Fig 5C and 5D**). To check if NbSGS3 has any effects on YFP-ATG8a accumulation, YFP-ATG8a accumulation levels were compared by Western blot between co-expression of NbSGS3 with YFP-ATG8a or with empty vector and result showed that YFP-ATG8a accumulation level were similar between the two treatments (**S8 Fig**), indicating that NbSGS3 has no negative effect on YFP-ATG8a accumulation. To further assess induction of autophagy when NbCaM and NbSGS3 are co-expressed, transmission electron microscopy (TEM) was used to monitor autophagic activity. Co-expression of NbCaM and NbSGS3 induced a 4-fold greater number of double-membrane structures typical of autophagosomes in the cytoplasm, as compared to expression of NbSGS3 or NbCaM alone (**Fig 5E and 5F**). Taken together, these results indicate that NbCaM and NbSGS3 are likely degraded by autophagy after they form a complex.

### The *N*. *benthamiana* PI3K complex is responsible for NbCaM-mediated NbSGS3 degradation and is necessary for the maximal infection of geminivirus associated with betasatellite

**Fig 6:**
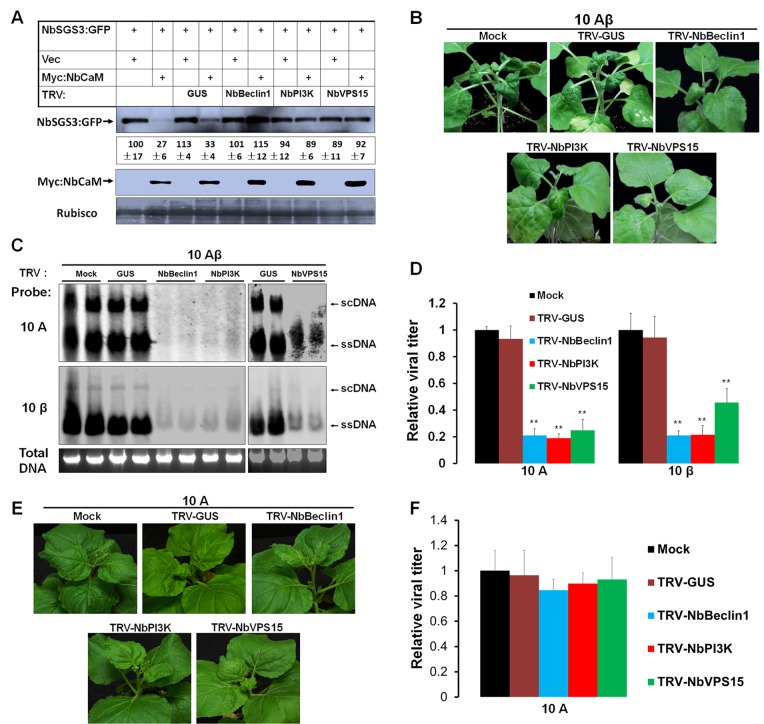
Silencing of *NbBeclin1*, *NbPI3K* or *NbVPS15* leads to inhibition of NbSGS3 degradation mediated by NbCaM and reduces the infection of geminivirus associated with betasatellite. **(A)** Silencing of *NbBeclin1*, *NbPI3K* or *NbVPS15* inhibits NbCaM-mediated NbSGS3 degradation. *N*. *benthamiana* plants were first inoculated with TRV vectors carrying partial fragments of *GUS*, *NbBeclin1*, *NbPI3K* or *NbVPS15* at the 4–5 leaf stage. Plants were then infiltrated at 21 dpi with NbSGS3:GFP plus Vec or NbSGS3:GFP plus Myc:NbCaM as indicated (top chart). Total protein was extracted from infiltrated leaves at 2 dpi, and GFP and Myc antibodies used in Western blot analyses. Coomassie brilliant blue staining of Rubisco large subunit was used as a loading control. A representative Western blot from at least three independent experiments is shown. NbSGS3:GFP protein bands were quantified using Image J software, and the protein level in leaves co-expressing NbSGS3:GFP and empty vector (Vec) in no-TRV infected plants was set at 100. Values represent the average level of protein ± standard deviation (SD). **(B)** Symptoms of mock, TRV-GUS and *NbBeclin1*, *NbPI3K* or *NbVPS15*-silenced plants infected by 10Aβ at 14 dpi. Systemic leaves of *NbBeclin1*, *NbPI3K* or *NbVPS15*-silenced plants at 7 dpi were then infected with TYLCCNV+TYLCCNB (10Aβ). (**C**) Southern blots of 10Aβ DNA accumulation using 30 μg of total DNA isolated from systemic leaves of six plants shown in (**B**) at 14 dpi. The agarose gel was stained with ethidium bromide as a loading control. Viral single-stranded DNA (ssDNA) and supercoiled DNA (scDNA) are indicated. (**D**) Relative 10A and 10β DNA levels in plants shown in (C) normalized to 25S rRNA that served as an internal plant genomic DNA control. The upper newly infected leaves were harvested and 100 ng total DNA used for relative quantitative PCR. The level of 10A or 10β DNA in mock-treated plants is arbitrarily set as 1. Values represent the mean ± standard deviation (SD) (n = 9). Double asterisks indicate a highly significant difference (p<0.01) between mock-treated and *ATG*s-silenced plants (student’s *t* test). **(E)** Symptoms of mock, TRV-GUS and *NbBeclin1*, *NbPI3K* or *NbVPS15*-silenced plants infected by 10A at 14 dpi. (**F**) Relative 10A DNA levels in plants shown in (E) normalized to 25S rRNA that served as an internal plant genomic DNA control. The upper newly infected leaves were harvested and 100 ng total DNA were used for relative quantitative PCR. The level of 10A DNA in mock-treated plants is arbitrarily set as 1. Values represent the mean ± standard deviation (SD) (n = 9).

To further understand the involvement of autophagy in NbCaM-mediated NbSGS3 degradation, the phosphatidylinositol 3-kinase (PI3K) complex containing Beclin1/VPS30/ATG6, PI3K/VPS34 and VPS15, which form phagophore to initiate autophagy [31], were analyzed. Predicted cDNA and protein sequences for *Beclin1*, *PI3K*, and *VPS15* were identified in *N*. *benthamiana (https://solgenomics.net/tools/blast/*) through sequence similarity to homologs in *A*. *thaliana* and *N*. *tabacum* [29, 46, 47]. Partial-length cDNA sequences were isolated using *N*. *benthamiana* cDNA and cloned into a *Tobacco rattle virus* (TRV)-based VIGS vector. *N*. *benthamiana* seedlings were agroinfiltrated with recombinant TRV vectors carrying partial fragments of *NbBeclin1*, *NbPI3K* and *NbVPS15*, respectively to induce silencing of each gene. Silencing of these genes in *N*. *benthamiana* plants did not result in any distinct developmental defects in systemic leaves (**S9A Fig**), when compared to TRV-GUS-infected plants (negative controls). At 14 dpi, in plants infiltrated with *ATG*-silencing vectors, mRNA levels of each *ATG* were reduced by approximately 80% as compared to negative controls (infiltrated with TRV-GUS or mock) (**S9B Fig**). Newly formed upper leaves in *ATG*-silenced, TRV-GUS-treated or mock plants (no TRV infection) were infiltrated with NbSGS3:GFP and empty vector (Vec) or NbSGS3:GFP and Myc:NbCaM at 21 dpi. Two days later, the newly infiltrated leaf tissue was examined by confocal microscopy to compare the fluorescence strength of NbSGS3:GFP. Western blot analysis was performed to determine levels of NbSGS3:GFP. In mock or TRV-GUS-treated plants, overexpression of NbCaM decreased the fluorescence intensity of NbSGS3:GFP (**S10 Fig**) and reduced the accumulation of NbSGS3:GFP 3-fold (**Fig 6A**). Silencing either *Beclin1*, *PI3K* or *VPS15* blocked degradation of NbSGS3 as determined by reduced levels of NbSGS3:GFP protein in infiltrated leaf patches (**Fig 6A**). These results demonstrate that the autophagy genes *Beclin1*, *PI3K* and *VPS15*, which constitute the PI3K complex, are required for NbCaM-mediated degradation of NbSGS3.

TYLCCNB-encoded βC1 up-regulates NbCaM to suppress RNA silencing and promote viral infection [10]. Given that NbSGS3 has an important role in defense against geminiviral infection and NbCaM-mediated NbSGS3 degradation appears to be dependent on the autophagy pathway, we next examined whether ATGs were also involved in geminivirus infection. Plants silenced for *NbBeclin1*, *NbPI3K* or *NbVPS15* at 7 dpi were inoculated with equal amounts of TYLCCNV and its betasatellite (10Aβ) and symptoms induced by 10Aβ in mock, TRV-GUS-treated or *ATG*s-silenced plants were observed. Infection induced by 10Aβ in TRV-GUS-treated plants showed leaf curling symptoms similar to those observed in mock plants at 14 dpi. In contrast, *NbBeclin1*, *NbPI3K* or *NbVPS15*-silenced plants developed much milder symptoms with reduced leaf curling (**Fig 6B**). In agreement with these observations, Southern blot analysis of viral genomic DNA levels indicated almost undetectable amounts of viral DNA accumulation of both helper virus (10A) and betasatellite (10β) in *NbBeclin1*, *NbPI3K* or *NbVPS15*-silenced plants (**Fig 6C**). qPCR analysis of 10A and 10β DNA levels in the upper emerged infected leaves also showed a significant reduction in viral DNA levels in *NbBeclin1*, *NbVPS15* or *NbPI3K*-silenced plants as compared to TRV-GUS-treated or mock plants (**Fig 6D**). These findings suggest that the PI3K complex is necessary for maximal symptom development and viral DNA accumulation, consistent with their role in the degradation of NbSGS3.

To determine whether these observations extend to geminivirus that lacks an associated betasatellite, mock, TRV-GUS-treated or *ATG*s-silenced plants were also inoculated with equal amounts of TYLCCNV alone (10A). No obvious viral symptom and viral DNA accumulation difference were observed in among mock, TRV-GUS-treated, and *NbBeclin1*, *NbPI3K* or *NbVPS15*-silenced plants at 14 dpi (**Fig 6E and 6F).** Similarly, the deficiency of *NbBeclin1*, *NbPI3K* or *NbVPS15* also had no obvious effect on the infectivity and viral DNA accumulations of *Tomato leaf curl Yunnan virus* (TLCYnV) and *Tobacco curly shoot virus* (TbCSV) without betasatellite (**S11 Fig**). These data indicate that the proviral role of autophagy in geminivirus biology depends on the presence of betasatellite.

## Discussion

In plants, RNA silencing is a major defense mechanism against foreign genes or viral invasion [2, 3]. As a counter defensive strategy, plant viruses have evolved VSRs as potent molecular weapons to counteract antiviral RNA silencing by interacting with key components of the cellular RNA silencing pathway, such as binding long or short dsRNA duplex, interacting with or disrupting AGOs, DCLs, RDRs and their functional partners, or interfering with the assembly of RISC [1, 15]. The function of calmodulin-like protein is still in dispute and its suppression mechanism is not clear. To help clarify the mechanism by which calmodulin-like protein suppresses RNA silencing, we showed that NbCaM interacts with NbSGS3 in the Y2H and BiFC systems, but not with NbRDR6 (**Fig 1**).

AtSGS3 localizes to cytoplasmic granules (SGS3/RDR6-bodies), where RDR6-mediated dsRNA synthesis is thought to occur [13]. NbSGS3 also localizes to SGS3/RDR6-bodies, along with NbRDR6 (**Fig 2D**), indicating that SGS3/RDR6-bodies are likely conserved among different plant species. However, the domains that are necessary for localization of NbSGS3 and AtSGS3 appear to be different. We showed that the ZF and CC domains are required for NbSGS3:GFP localization, but the XS and CC domains are necessary for AtSGS3:GFP localization [13].

As the partner of RDR6, SGS3 can bind the 5’ overhang of dsRNAs and may prevent degradation of these dsRNAs, alter their localization, and/or recruit them as templates for dsRNA synthesis process [11, 14, 48]. Therefore, it is not surprising that SGS3 is targeted by several VSRs, including the V2 protein of *Tomato yellow leaf curl virus* (TYLCV) [49], p2 of *Rice stripe virus* (RSV) [50], the VPg protein of *Potato virus A* (PVA) [51], TGBp1 of *Planta goasiatica mosaic virus* (PlAMV) [52]. Our data showed that NbSGS3 is also a target of the endogenous RNA silencing suppressor, NbCaM. First, NbCaM interacted with NbSGS3 in yeast and in planta (**Fig 1**). Second, deletion mutants lacking the EFI and EFII domains lost the ability to interact with NbSGS3, and failed to suppress GFP-induced S-PTGS (**Fig 3F and 3G**). Finally, overexpression of NbCaM led to a reduced accumulation of NbSGS3 in granules and promoted its degradation (**Fig 4**). Our data also demonstrate that the interaction between NbCaM and NbSGS3 is required for the suppressor activity of NbCaM. Meanwhile, the ZF and CC domains of NbSGS3, which are necessary for localization to the SGS3/RDR6-bodies, are also required for the interaction with NbCaM. This suggests that these two domains play an important role in inducing RNA silencing.

Autophagy has been reported to play a central role in several physiological and developmental responses in plants, such as nutrient recycling, seed development and germination, nitrogen or carbon deprivation [44, 45, 53, 54], and plant immunity and programmed cell death [29, 55]. A recent study showed that NtCaM could mediate degradation of the dsRNA binding VSR 2b via the autophagy-like protein degradation pathway [21]. We found that overexpression of NbCaM induced degradation/reduction of NbSGS3 when co-expressed (**Fig 4**). Inhibition of autophagy (3-MA or E64d, **S5 Fig**) or the 26S proteasome (**S6 Fig**) did not have obvious effects on the accumulation of GFP, NbCaM:GFP or NbSGS3:GFP, when expressed alone. In contrast, 3-MA-treatment blocked degradation of both NbCaM and NbSGS3, when co-expressed and led to an increase in the level of their corresponding proteins (**Fig 5A and 5B**). This phenomenon suggests that individual expression of either NbCaM or NbSGS3 does not trigger autophagy-mediated degradation, but that interaction between NbCaM and NbSGS3 activates the autophagy system, possibly via recruitment of some ATG proteins. Indeed, degradation was blocked when *NbBeclin1*, *NbPI3K* or *NbVPS15*, which constitute the PI3K complex, was silenced. In addition, when *NbBeclin1*, *NbPI3K* or *NbVPS15* was knocked-down, TYLCCNV and TYLCCNB (10Aβ) was unable to infect plants efficiently, showing very mild viral symptoms and almost undetectable viral DNA levels (**Fig 6A–6D**), suggesting a necessary role for the PI3K complex in viral replication and systemic infection. It is worthy to note that the knock-down of *NbBeclin1*, *NbPI3K* or *NbVPS15* has no effect on the infection of this geminivirus that lacks an associated betasatellite (10A) (**Fig 6E and 6F**). These data indicate that the proviral role of autophagy in geminivirus biology depends on the presence of betasatellite, which is consistent with the fact that 10Aβ not 10A significantly up-regulates NbCaM expression and induces severe symptoms [10, 62]. Similarly, the other two geminiviruses in the absence of betasatellite showed no obvious differences in their infectivity in mock and *ATGs*-silenced plants (**S11 Fig**). Therefore, it seems that geminiviruses that lack an associated betasatellite fail to utilize autophagy factors to defend NbRDR6/NbSGS3-dependent resistance.

RDR6 and SGS3 play important roles in RNA silencing, and their expression can be effectively fine-tuned. For NbCaM to be an effective negative regulator of RNA silencing, it needs to repress both NbRDR6 and NbSGS3. In support of this, our previous study showed that NbCaM suppressed *NbRDR6* mRNA levels [10]. Plant calmodulin-like proteins can directly bind to DNA and function as transcription factors (TF) to activate or suppress a target gene’s expression [56]. For example, an *Arabidopsis* calmodulin-binding transcription factor CAMTA3 functions as a suppressor of defense response and can activate gene expression by directly binding to promoters of suppressed genes or suppressing gene expression by activating expression of a repressor [57]. We found that NbCaM could activate expression of a reporter gene in yeast cells (**S12 Fig**), which indicates that NbCaM may also function as a TF. It is therefore possible that NbCaM suppresses *NbRDR6* via binding to a promoter element and repressing its expression. However, further study is necessary to determine the mechanism of NbCaM suppression of *NbRDR6* expression.

**Fig 7:**
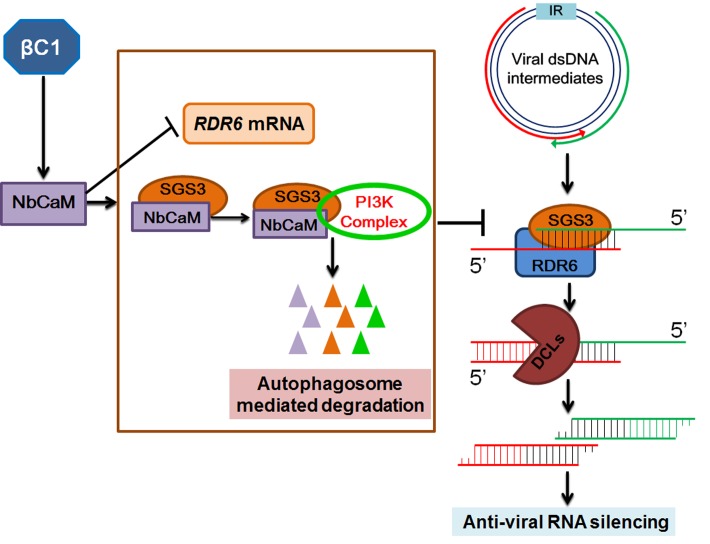
A working model summarizing the roles of the endogenous suppressor NbCaM in regulation of PTGS and geminivirus infection. The replication intermediates produced during geminivirus infection serve as templates for bidirectional transcription, and the resulting convergent transcripts induce RNA silencing. The dsRNAs with 5’ overhangs are further recognized by SGS3 and then converted into longer dsRNAs by RDR6, which amplifies RNA silencing and processes secondary siRNAs targeted against geminivirus infection. As a cellular negative regulator of RNA silencing, NbCaM could repress expression of *RDR6* by an unidentified mechanism. Alternatively, the betasatellite encoded βC1 up-regulates NbCaM expression and NbCaM interacts with NbSGS3 to mediate degradation of NbSGS3 via the autophagy pathway. This then promotes efficient viral invasion by suppression of the host anti-viral RNA silencing machinery.

Together with previous studies, we conclude that the cellular suppressor NbCaM not only suppresses *NbRDR6* transcription, but also interacts with the RNA silencing component NbSGS3 and mediates its degradation by recruiting autophagy factors. Geminivirus betasatellite appears to utilize NbCaM in suppression of plant antiviral defenses, which leads to successful viral infection and multiplication (**Fig 7**).

## Materials and methods

### Plant materials and growth conditions

*N*. *benthamiana* seedlings were placed in soil and incubated in an insect-free growth chamber at 25°C and 60% relative humidity under a 16 h light/8 h dark photoperiod. The transgenic H2B-RFP line was gift of Michael M. Goodin (University of Kentucky, USA).

### Cloning of full-length NbSGS3 cDNA and plasmid constructs

The tobacco *NtSGS3* and tomato *SlSGS3* sequences were used to identify orthologous sequences from available *N*. *benthamiana* ESTs. BLAST searches revealed high homology between SGS3 from tobacco and *N*. *benthamiana* (https://blast.ncbi.nlm.nih.gov/Blast.cgi?CMD=Web&PAGE_TYPE=BlastHome). Primers designed to anneal to conserved sequences in the 5' and 3' untranslated regions of tobacco SGS3 were used to amplify the coding region of *N*. *benthamiana* SGS3 by reverse transcription PCR (RT-PCR). Amplification with primer pairs NbSGS3-cds-F/NbSGS3-cds-R yielded a specific product of approximately 1900-bp, which was cloned into pMD18-T (TaKaRa, Dalian, China) and then sequenced. Detailed primer information is given in **S1 Table**. The full-length gene of *NbSGS3* was deposited in GenBank under the accession number KJ190939. The full-length *NbSGS3* was amplified using primer pair NbSGS3-F/NbSGS3-R and cloned into the binary vectors pCHF3-Flag, pCHF3-GFP or pCHF3-RFP between the *Bam*HI and *Sal*I sites. The resulting plasmids (pCHF3-35S-NbSGS3:Flag, pCHF3-35S-NbSGS3:GFP or pCHF3-35S-NbSGS3:RFP) were used for overexpression in transgenic plants or transient agroinfiltration assays using the CaMV 35S promoter. *NbSGS3* was introduced into the 2YN or 2YC BiFC vectors between the *Pac*I and *Asc*I sites to generate 2YN-NbSGS3 or 2YC-NbSGS3 for BiFC analysis. Mutants of NbSGS3 were generated by overlapping PCR [58], using the corresponding primer pairs given in **S1 Table** and cloned into the 2YN, 2YC and pCHF3-GFP vectors.

To construct a TRV-based recombinant VIGS vector containing *NbBeclin1*, *NbPI3K* or *NbVPS15*, a partial fragment of each gene was generated by PCR amplification using the respective primer pair and cloned into the pTRV2 vector (a kind gift of Yule Liu) [59] using the restriction enzyme sites listed in **S1 Table**.

The coding sequence of NbCaM was amplified by PCR from *N*. *benthamiana* and introduced into the vectors pCHF3-Flag, pCHF3-GFP, 2YN or 2YC using the primers and restriction enzyme sites listed in **S1 Table**. pCHF3-based vectors were used for transient expression of *NbCaM* in *N*. *benthamiana* leaves. Construction of NbCaM mutants by overlapping PCR was similar to that described for NbSGS3 mutants [58], using the corresponding primers described in **S1 Table**. For the pCHF3-NbRDR6:GFP construct, the coding sequence of NbRDR6 was amplified by PCR from *N*. *benthamiana* and introduced into pCHF3-GFP between the *Sma*I and *Sal*I sites using the corresponding primers described in **S1 Table.** For 2YN-NbRDR6 or 2YC-NbRDR6, the NbRDR6 coding sequence was introduced into the 2YN or 2YC BiFC vectors between the *Pac*I and *Asc*I sites. 2YN-P3N-PIPO and 2YC-P3N-PIPO have been described previously [40].

The pBA-Flag-Myc4:NbSGS3 (Myc4:NbSGS3), pEarleygate104:NbSGS3 (YFP:NbSGS3), pEarleygate101:NbSGS3 (NbSGS3:YFP), pBA-Flag-Myc4:NbCaM (Myc4:NbCaM) or pEarleygate104:NbATG8a (YFP:NbATG8a) construct was generated using gateway technology (Invitrogen, Burlington, Ontario, Canada) with the corresponding primer pairs given in **S1 Table**.

GenBank accession numbers for the genes analyzed in this study are as follows: *NbSGS3* (KJ190939), *NtSGS3* (NM_001325691), *SlSGS3* (NM_001247782), *AtSGS3* (NM_122263), *NbRDR6* (AY722008), *NbBeclin1* (AY701316), *NbPI3K* (AY701317), *NbVPS15* (KU561371) and *NbATG8a* (KX120976).

### Y2H, BiFC, subcellular localization

Two-hybrid screen experiments to assess the different interactions between NbCaM, NbSGS3 and NbRDR6 in yeast were performed as described previously [60]. For BiFC and subcellular localization experiments fluorescence were examined in epidermal cells of 1–2 cm^2^ leaf explants by confocal microscopy (Leica TCS SP5, Mannheim, Germany) from 36 h to 72 h post infiltration as described [60].

### Viral inoculation and agroinfiltration

For geminivirus agroinoculation, equal volumes of individual *A*. *tumefaciens* cultures at an OD_600_ of 1 were mixed prior to inoculations. Infectious virus clones, including TYLCCNV (pBinPLUS-Y10-1.7A), TYLCCNV/TYLCCNB (pBinPLUS-Y10-1.7A+Y10β), TbCSV (pBinPLUS-Y35-1.9A) and TLCYnV (pBinPLUS-Y194-1.4A) have been described previously [61–63]. *Agrobacterium* cultures carrying infectious virus clone(s) were infiltrated into *N*. *benthamiana* leaves and inoculated plants were photographed with a Canon 400D digital camera at different time periods.

For the TRV-VIGS assay, *Agrobacterium* cultures harboring pTRV1 and pTRV2-VIGS (TRV2-GUS, TRV2-NbBeclin1, TRV2-NbPI3K or TRV2-NbVPS15) were resuspended in infiltration buffer (10 mM MgCl_2_, 10 mM MES (pH5.6), and 100 μM acetosyringone) and mixed at a 1:1 ratio. After a 3 h incubation at room temperature, the mixed *Agrobacterium* cultures were infiltrated into leaves of *N*. *benthamiana* plants at the 5–6 leaf stage. A silenced phenotype appeared in the upper leaves at 2 weeks post infiltration.

### DNA isolation, DNA blot hybridization and qPCR

Total DNA was extracted from infected plants using the CTAB method [64], and DNA blot hybridization performed to assess viral DNA accumulation essentially as described [65]. Total DNA electrophoresed through agarose gels was stained with ethidium bromide to ensure equal loading. After denaturation and neutralization, total DNA was transferred to Hybond N^+^ nylon membranes (GE Healthcare, Pittsburgh, PA, USA) by capillary transfer. Membranes were hybridized at 45°C to specific probes labeled with digoxigenin (Roche Diagnostics, Rotkreuz, Switzerland). Viral DNA levels were determined by qPCR using specific primers (**S1 Table**) and normalized to 25S RNA as an internal genomic DNA control [66].

### RNA extraction and RT-qPCR analysis

Total RNA was isolated from virus-infected plants and different plant organs using Trizol reagent (Invitrogen, Carlsbad, CA, USA). For RT-qPCR analysis, 1 μg total RNA was firstly treated with DNase I, and then the first strand cDNA was synthesized from the treated RNA by using Oligo(dT)12-18 primer and SuperScript III reverse transcriptase (Invitrogen) following the recommended protocol. All primer information used in RT-PCR was given in **S1 Table**, and the specific primer pairs for qRT-PCR were designed by Primer Premier 5 software [10].

### Immunoblotting

Total protein was extracted from infiltrated leaf patches as described previously [67]. Immunoblotting was performed with primary mouse monoclonal or rabbit polyclonal antibodies, followed by goat anti-mouse or anti-rabbit secondary antibody conjugated to horseradish peroxidase (Bio-Rad, Hercules, CA, USA). The GFP polyclonal antibody was obtained from Abcam (Massachusetts, US), and the Myc monoclonal antibody was obtained from Sigma (Los Angeles, CA, USA). Blotted membranes were washed thoroughly and visualized using chemiluminescence according to the manufacturer’s protocol (ECL; GE Healthcare).

### Chemical treatments and TEM

PBS buffer containing 2% DMSO (control) or an equal volume of DMSO with 10 mM 3-MA and 100 uM E64d (Sigma) for inhibition of autophagy, or 100 μM MG132 (Sigma) for inhibition of the 26S proteasome was infiltrated into leaves 16 h before samples were collected. For TEM observation, detailed information has been described previously [46]. Vec and NbSGS3, or NbCaM, or NbSGS3 +Vec or NbSGS3 +NbCaM -infiltrated leaves pretreated with 10 mM 3-MA for 8 h, and then were cut into small pieces (1 mm × 4 mm). The sampled tissues were fixed in 2.5% glutaraldehyde and 1% osmium tetroxide (both in 100 mM phosphate buffer (PB), pH 7.0). The samples were then post-fixed in OsO4, dehydrated in ethanol, and then embedded in Epon 812 resin as instructed by the manufacture (SPI-EM, Division of Structure Probe, Inc., West Chester, USA). Ultrathin sections (70 nm) were cut with a diamond knife from the embedded tissues using the Ultracut E Ultramicrotome (Reichart-Jung, Vienna, Austria) and were collected on 3-mm copper (mesh) grids, and then stained with uranyl acetate and lead citrate before final examination under an electron microscope, Model JEM-1230.

## Supporting information

S1 Table Primers used in plasmid construction and other experiments in this study. (DOC)

S1 Fig Interaction between NbSGS3 and NbRDR6, self-interaction of NbSGS3, and confirmation of protein expression in co-transformed yeast cells by Western blot (WB). **(A, B)** Yeast two-hybrid assays for NbSGS3 and NbRDR6 (**A)**, or NbSGS3 and NbSGS3 (**B**). Serial 10-fold dilutions of yeast cells were made as indicated. Cells co-transformed with AD-T7-T+BD-T7-53 serve as positive controls; cells co-transformed with AD-T7-T+BD-T7-Lam, or with the empty vectors pGBKT7 (BD) and pGADT7 (AD) are negative controls. BD, GAL4 DNA binding domain; AD, GAL4 activation domain. **(C-F)** Protein expression in co-transformed yeast cells was confirmed by Western blot. Total protein was extracted from yeast cells transformed with the indicated plasmids. AD vector is HA-tagged and BD vector is Myc-tagged. Antibodies against HA- (WB:HA) and Myc- (WB:Myc) tags were used. Ponceau staining of total protein served as a loading control.
(TIF)

S2 Fig No interaction between NbCaM and NbRDR6, or NbSGS3 and P3N-PIPO. BiFC assays in *N*. *benthamiana* leaves expressing NbCaM and NbRDR6, or NbSGS3 and P3N-PIPO in H2B-RFP transgenic *N*. *benthamiana* leaves at 48 hours post infiltration (hpi). Bars = 50 μm. No YFP fluorescence was detected.
(TIF)

S3 Fig Subcellular localization of NbSGS3 in *Nicotiana benthamiana*. (**A)** Micrographs showing cells from protoplasts, which were prepared from *N*. *benthamiana* leaves transformed with 20 μg of 35S:NbSGS3:GFP plasmid DNA using polyethylene glycol (PEG)-mediated transformation, and micrographs taken 36 h after transformation. Bars = 10 μm. GFP fluorescence was detected in punctate spots in the cytoplasm. (**B**) Micrographs showing cells from leaves of H2B-RFP transgenic *N*. *benthamiana* expressing YFP:NbSGS3. Yellow fluorescence was detected from YFP:NbSGS3 (Green). Red fluorescence showed H2B-RFP as a nuclear marker. Bars = 50 μm.
(TIF)

S4 Fig No co-localization between NbSGS3:GFP and mitochondria tracker, golgi or peroxisome marker in *Nicotiana benthamiana*. (**A**) Micrographs showing cells from *N*. *benthamiana* leaves firstly infiltrated with NbSGS3:GFP and then stained with mitochondria tracker red (Mito-Tracker:Red) at 48 hpi. (**B**) Micrographs showing cells co-expressing NbSGS3:GFP and golgi marker Man49:mCherry. (**C**) Micrographs showing cells co-expressing NbSGS3:GFP and peroxisomes marker dsRED:SKL. Bars = 50 μm.
(TIF)

S5 Fig **The effects of autophagy inhibitors 3-MA or E64d on the accumulation of GFP (A), NbCaM:GFP (B) or NbSGS3:GFP (C) protein detected by Western blot using GFP antibody**. No treatment (-), DMSO, 3-MA (10 mM) or E64d (100 uM) treated samples were harvested from plants agroinfiltrated with GFP, NbCaM:GFP or NbSGS3:GFP. Ponceau staining of Rubisco large subunit was used as a loading control.
(TIF)

S6 Fig Inhibition of the 26S-proteasome did not impact NbSGS3:GFP, NbCaM:GFP, or GFP protein levels. The effects of the 26S-proteasome inhibitor MG132 on accumulation of NbSGS3:GFP, NbCaM:GFP or GFP proteins were measured by WB using a GFP antibody (WB:GFP). DMSO (DM), or an equal volume of MG132 (100 μM) was infiltrated 16 h before the samples were harvested. Ponceau staining was used as a loading control.
(TIF)

S7 Fig Effect of the autophagy inhibitors 3-MA and E64d on the accumulation of NbSGS3:GFP when expressed alone or together with Myc:NbCaM at 2 dpi. Samples were analyzed by Western blot using GFP antibody. *Agrobacterium* culture mixtures containing a plasmid capable of expressing NbSGS3:GFP alone, or together with Myc:NbCaM are indicated. DMSO (control), 3-MA (10 mM) or E64d (100 uM) was infiltrated into *Nicotiana benthamiana* leaves 32 h after being agroinfiltrated with NbSGS3:GFP alone or together with Myc:NbCaM constructs. Samples were harvested from 16 h later. Ponceau staining of Rubisco large subunit was used as a loading control.
(TIF)

S8 Fig The effect of NbSGS3 on YFP:ATG8a protein accumulation analyzed by Western blot. Samples were harvested from plants agroinfiltrated with YFP:ATG8a and Myc:NbSGS3 or empty vector (Vec) at 48 hours post infiltration. GFP (WB:GFP) and Myc (WB:Myc) antibodies were used in Western blot analysis and Coomassie brilliant blue staining of Rubisco large subunit was used as a loading control.
(TIF)

S9 Fig Phenotypes and the RNA silencing efficiency of the *ATG*-deficient plants. **(A)** The phenotype in mock, TRV-GUS, TRV-NbBeclin1, TRV-NbPI3K, or TRV-NbVPS15-treated plants at 21 dpi. Partial fragments of *NbBeclin1*, *NbPI3K* and *NbVPS15* were cloned into RNA2 of the TRV VIGS vector. *N*. *benthamiana* plants at the 4–5 leaf stage were infiltrated with *Agrobacterium* cultures carrying an empty vector (mock), pTRV1 and pTRV2-GUS, or pTRV1 and pTRV2-VIGS. **(B)** Silencing of the indicated *ATG* genes (*NbBeclin1*, *NbPI3K* or *NbVPS15*) was confirmed in newly emerged leaves 14 dpi by RT-qPCR. Values for the *ATG* genes in TRV-GUS infected plants were arbitrarily set 1. Double asterisks indicate a highly significant difference (p<0.01) between TRV-GUS infected plants and TRV-ATGs infected plants (student’s *t* test).
(TIF)

S10 Fig Micrographs showing cells expressing NbSGS3:GFP with Vec or Myc:NbCaM in mock or TRV-GUS treated plants. The newly leaves of mock (no TRV infection), or TRV-GUS-treated *N*. *benthamiana* plants were infiltrated with NbSGS3:GFP and empty vector (Vec) or NbSGS3:GFP and Myc:NbCaM at 21 dpi. Infiltrated leaves were examined at 48 hpi. Bars represent 50 μm.
(TIF)

S11 Fig Silencing of *NbBeclin1*, *NbPI3K* or *NbVPS15* fails to affect the infection of TLCYnV or TbCSV. **(A, B)** Symptoms of mock, TRV-GUS and *NbBeclin1*, *NbPI3K* or *NbVPS15*-silenced *N*. *benthamiana* plants infected by TLCYnV (A) or TbCSV (B) at 14 dpi. (**C**) Relative TLCYnV or TbCSV DNA accumulation levels in plants shown in (A, B) normalized to 25S rRNA that served as an internal plant genomic DNA control. The upper newly infected leaves were harvested and 100 ng total DNA were used for relative quantitative PCR. The level of TLCYnV or TbCSV DNA in mock plants is arbitrarily set as 1. Values represent the mean ± standard deviation (SD) (n = 9).
(TIF)

S12 Fig The transcriptional activation activity of NbCaM. Yeast cells carrying AD+BD:NbCaM, BD alone (negative control) or AD+BD-AC2 (positive control) were cultured on the selective synthetic complete medium as indicated and then were photographed after 3 days. AC2 was cloned from *Mungbean yellow mosaic virus*.
(TIF)
