# Supplementary material for: A calmodulin-like protein suppresses RNA silencing and promotes geminivirus infection by degrading SGS3 via the autophagy pathway in Nicotiana benthamiana
Source: PLoS Pathog. 2017 Feb 17;13(2):e1006213. doi: 10.1371/journal.ppat.1006213 (PMC5333915; doi:10.1371/journal.ppat.1006213)
Supplement: S1 Table — (DOC) [file ppat.1006213.s001.doc]

**S1 Table. Primers used in plasimd construction and other experiments in this study.**

| Primer Name | Primer Sequence (5’—3’) | Purpose |
| --- | --- | --- |
| NbSGS3:Flag-F | CGGGATCCATGAGTTCAAGCAAAGGGGTTG | pCHF3-NbSGS3: Flag, transient expression and transgene analysis |
| NbSGS3:Flag-R | GCGTCGACTTGAGATTGCTCTGGGGAG |  |
| NbSGS3-AD/BD-F | CGGAATTCATGAGTTCAAGCAAAGGGGTTG | pGADT7/pGBKT7-NbSGS3, Y2H analysis |
| NbSGS3-AD/BD-R | CGGGATCCGCTATTGAGATTGCTCTGGGGAG |  |
| NbCaM-AD/BD-F | CGGAATTCATGTGCATGGAATCAGT | pGADT7/pGBKT7-NbCaM, Y2H analysis |
| NbCaM-AD/BD-F | CGGGATCCTTAACTTGTCATCATAGC |  |
| NbRDR6-AD/BD-F | TCCCCCGGGGATGGGATCAGAGGGCTCTG | pGADT7/pGBKT7-NbRDR6, Y2H analysis |
| NbRDR6-AD/BD-R | GCGTCGACGTCATATATTGTCAACAAGATACCTTC |  |
| NbSGS3-BiFC-F | CCTTAATTAACATGAGTTCAAGCAAAGGGGTTG | p2YN/p2YC-NbSGS3, BiFC analysis |
| NbSGS3-BiFC-R | AGGCGCGCCCTTGAGATTGCTCTGGGGAG |  |
| NbCaM-BiFC-F | CCTTAATTAACATGTGCATGGAATCAGTTTCTG | p2YN/p2YC-NbCaM, BiFC analysis |
| NbCaM-BiFC-R  NbRDR6-BiFC-F  Nb RDR6-BiFC-R | AGGCGCGCCCACTTGTCATCATAGCTTTGAACTC  CCTTAATTAACATGGGATCAGAGGGCTCTG  AGGCGCGCCCTATATTGTCAACAAGATACCTTC | p2YN/p2YC-NbRDR6, BiFC analysis |
| GFP-F | CGGGATCCATGGTGAGCAAGG | pCHF3-GFP, subcellular localization analysis |
| GFP-R | GCCTGCAGTTACTTGTACAGCTC |  |
| NbCaM:GFP-F | GAGCTCATGTGCATGGAATCA | pCHF3-NbCaM:GFP, subcellular localization analysis |
| NbCaM:GFP-R | GGATCCACTTGTCATCATAGC |  |
| NbSGS3:GFP/RFP/Flag-F | CACGAGCTCATGAGTTCAAGCAAAGGGGTTG | pCHF3-NbSGS3:GFP/RFP/Flag, subcellular localization and transient expression analysis |
| NbSGS3: GFP/RFP/Flag-R | GCTCTAGATTGAGATTGCTCTGGGGAG |  |
| RFP-F | GGGGTACCTCTAGAATGGCCTCCTCCGAGAAC | pCHF3-RFP, subcellular localization analysis |
| RFP-R | CGGGATCCGTCGACTTACAGGAACAGGTGGTG |  |
| NbCaM:Flag-F | GGGAGCTCATGTGCATGGAATCAGTT | pCHF3-NbCaM:Flag, transient expression analysis |
| NbCaM:Flag-R | GCGGATCCACTTGTCATCATAGCTTTG |  |
| NbRDR6:GFP-F | TCCCCCGGGGATGGGATCAGAGGGCTCTG | pCHF3-NbRDR6:GFP, transient expression analysis |
| NbRDR6:GFP-R | GCGTCGACGTATATTGTCAACAAGATACCTTC |  |
| NbSGS3/dZF/p-s/F | CCTTAATTAACGAGCTCATGGGTACTTCTGTTGTACCTCC | Cloning of NbSGS3-dZF into pCHF3:GFP, p2YN or p2YC vector |
| NbSGS3/dZF/XS/a/b/2R | AGGCGCGCCCGGATCCTTGAGATTGCTCTGGGGAGTAC |  |
| NbSGS3/dXS/CC/p/s/1F | CCTTAATTAACGAGCTCATGAGTTCAAGCAAAGGGGTTG | Cloning of NbSGS3-dXS/dCC into pCHF3:GFP, p2YN or p2YC vector |
| NbSGS3/dXS/over/1R | CATCATCTGCTTTGCTGGTATCTCACGCAGTTCTTC |
| NbSGS3/dXS/over/2F | GAAGAACTGCGTGAGATACCAGCAAAGCAGATGATG |
| NbSGS3/dCC/a/b/R | AGGCGCGCCCGGATCCATTTCCAACAGCTTCCTGGTATG |  |
| NbCaM/dX/p/s/F | CCTTAATTAACGAGCTCATGGTATTTACGTACTTTGACG | Cloning of NbCaM-dX into pCHF3:Flag, p2YN or p2YC vector |
| NbCaM/dX12/a/b/R | AGGCGCGCCCGGATCCACTTGTCATCATAACTTTG |  |
| NbCaM/dEF124/p/s/1F | CCTTAATTAACGAGCTCATGGAATCGGTTTCTGTACC | Cloning of NbCaM-dEF1 into pCHF3:GFP, p2YN or p2YC vector |
| NbCaM/dEF1/over/1R | CATCTCCGCCTCCTCCACCCTCTCTAACTCGCTACT |
| NbCaM/dEF1/over/2F | AGTAGCGAGTTAGAGAGGGTGGAGGAGGCGGAGATG |  |
| NbCaM/dEF2/over/1R | CAAGCTCTTAGGAGTAACCGTCAGTTCGCCTCCTAC | Cloning of NbCaM-dEFII into pCHF3:GFP, p2YN or p2YC vector |
| NbCaM/dEF2/over/2F | GTAGGAGGCGAACTGACGGTTACTCCTAAGAGCTTG |
| NbCaM/dEF4/a/b/R | A*GGCGCGCC*CGGATCCGTAGCCACTCCCCTCCATTTC | Cloning of NbCaM-dEFIV into pCHF3:GFP, p2YN or p2YC vector |
| NbSGS3-GW-F | GGGGACAAGTTTGTACAAAAAAGCAGGCTTCATGAGTTCAAGCAAAGGGGTTG | To make Gateway entry recombinant plasmid pDNOR221-NbSGS3 |
| NbSGS3-GW-R | GGGGACCACTTTGTACAAGAAAGCTGGGTCTTGAGATTGCTCTGGGGAG |  |
| NbCaM-GW-F | GGGGACAAGTTTGTACAAAAAAGCAGGCTTCATGTGCATGGAATCAGTTTCTG | To make Gateway entry recombinant plasmid pDNOR221-NbCaM |
| NbCaM-GW-R | GGGGACCACTTTGTACAAGAAAGCTGGGTCACTTGTCATCATAACTTTGAAC |  |
| NbATG8a-GW-F | GGGGACAAGTTTGTACAAAAAAGCAGGCTTCATGGCAAAGAGTTCATTCAAG | To make Gateway entry recombinant plasmid pDNOR221-NbATG8a |
| NbATG8a-GW-R | GGGGACCACTTTGTACAAGAAAGCTGGGTCCACCAAGTTAAAGTCCCC |  |
| TRV2-NbBeclin1-F | CGGGATCCGCCTTCTTCTTCATACAATGGCTC | TRV2- NbBeclin1, the silencing of *NbBeclin1* |
| TRV2- NbBeclin1-R | CCGCTCGAGGAGCTTTGGTCCAACTTTCCTG |  |
| TRV2-NbPI3K-F | CGGGATCCTGGCAACTGGACACGATGAGG | TRV2- NbPI3K, the silencing of *NbPI3K* |
| TRV2- NbPI3K-R | CCGCTCGAGGGCGGTGGAAAGGGCTTAGG |  |
| TRV2-NbVPS15-F | CGGGATCCGCTGAAGGTTCTTATTTTAATACTC | TRV2- NbVPS15, the silencing of *NbVPS15* |
| TRV2-NbVPS15-R | CCGCTCGAGTCGGGTACTAAGACGATCGTGCAG |  |
| NbGADPH-q-F | GCAGTGAACGACCCATTTATCTC | Relative qRT-PCR analysis of *NbGADPH* |
| NbGADPH -q-R | AACCTTCTTGGCACCACCCT |  |
| NbBeclin1-q-F | GACCTGCGTAAAGGAGTTTGCTGAC | Relative qRT-PCR analysis of *NbBeclin1* |
| NbBeclin1-q-R | CCAACAAACCAGTAGAGCACCCAC |  |
| NbPI3K-q-F | AGCTGTGCTGGTTACTCCGTCATC | Relative qRT-PCR analysis of  *NbPI3K* |
| NbPI3K -q-R | GTACTGACTTTCCGCTCCACCCATA |  |
| NbVPS15-q-F | ACCTGCCTTGATCATCCACATGTTTGG | Relative qRT-PCR analysis of  *NbVPS15* |
| q-25S-rRNA-F | ATAACCGCATCAGGTCTCCA | As an internal control for relative quantitative genomic PCR analysis |
| q-25S-rRNA-R | CCGAAGTTACGGATCCATTT |  |
| q-10A-F | TTAGAGATCGTCGTCCTAGTGG | Relative quantitative PCR analysis of TYLCCNV |
| q-10A-F | GCTCCTTACAAGCATATTGTCC |  |
| q-10b-F | ATACATCATACTCATCCCCTACATCTA | Relative quantitative PCR analysis of TYLCCNB |
| q-10b-R | ATTATCCCACCATTCGACTTCAACATT |  |
| q-TLCYnV-F | GGTGCGTCGCCGTCTGAACTTCG | Relative quantitative PCR analysis of TLCYnV |
| q-TLCYnV-R | CCAGTATGAGATACATCATGACGGGCC |  |
| q-TbCSV-F | TACGCCGCCGTCTCAACTTCGAC | Relative quantitative PCR analysis of TbCSV |
| q-TbCSV-R | CTTTACCTATATGCTGAATGTCATGTCTGG |  |
