# Supplementary figures and images for: A calmodulin-like protein suppresses RNA silencing and promotes geminivirus infection by degrading SGS3 via the autophagy pathway in Nicotiana benthamiana
Source: PLoS Pathog. 2017 Feb 17;13(2):e1006213. doi: 10.1371/journal.ppat.1006213 (PMC5333915; doi:10.1371/journal.ppat.1006213)

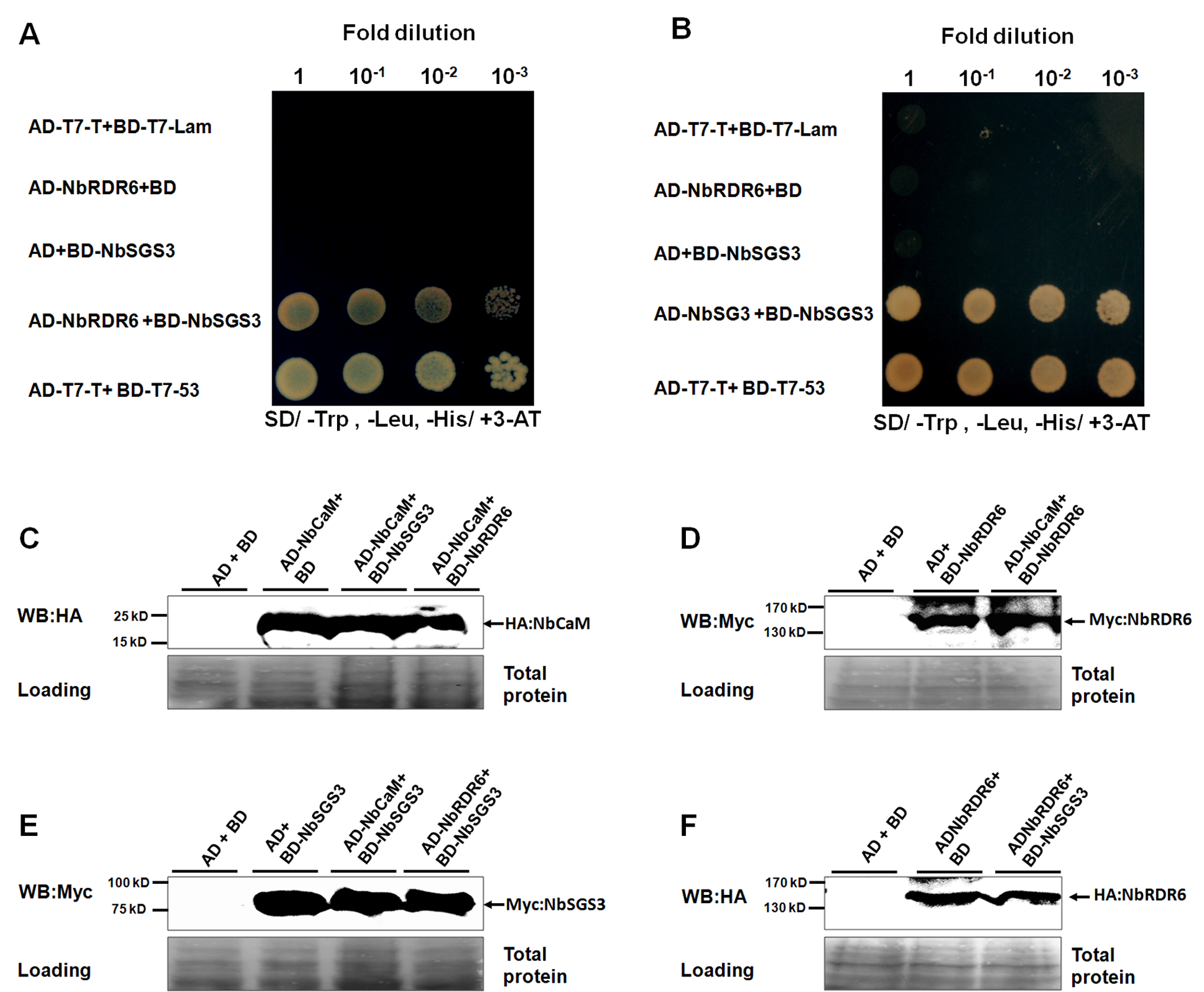

Supplement: S1 Fig — (A, B) Yeast two-hybrid assays for NbSGS3 and NbRDR6 (A), or NbSGS3 and NbSGS3 (B). Serial 10-fold dilutions of yeast cells were made as indicated. Cells co-transformed with AD-T7-T+BD-T7-53 serve as positive controls; cells co-transformed with AD-T7-T+BD-T7-Lam, or with the empty vectors pGBKT7 (BD) and pGADT7 (AD) are negative controls. BD, GAL4 DNA binding domain; AD, GAL4 activation domain. (C-F) Protein expression in co-transformed yeast cells was confirmed by Western blot. Total protein was extracted from yeast cells transformed with the indicated plasmids. AD vector is HA-tagged and BD vector is Myc-tagged. Antibodies against HA- (WB:HA) and Myc- (WB:Myc) tags were used. Ponceau staining of total protein served as a loading control. (TIF) [file ppat.1006213.s002.tif]

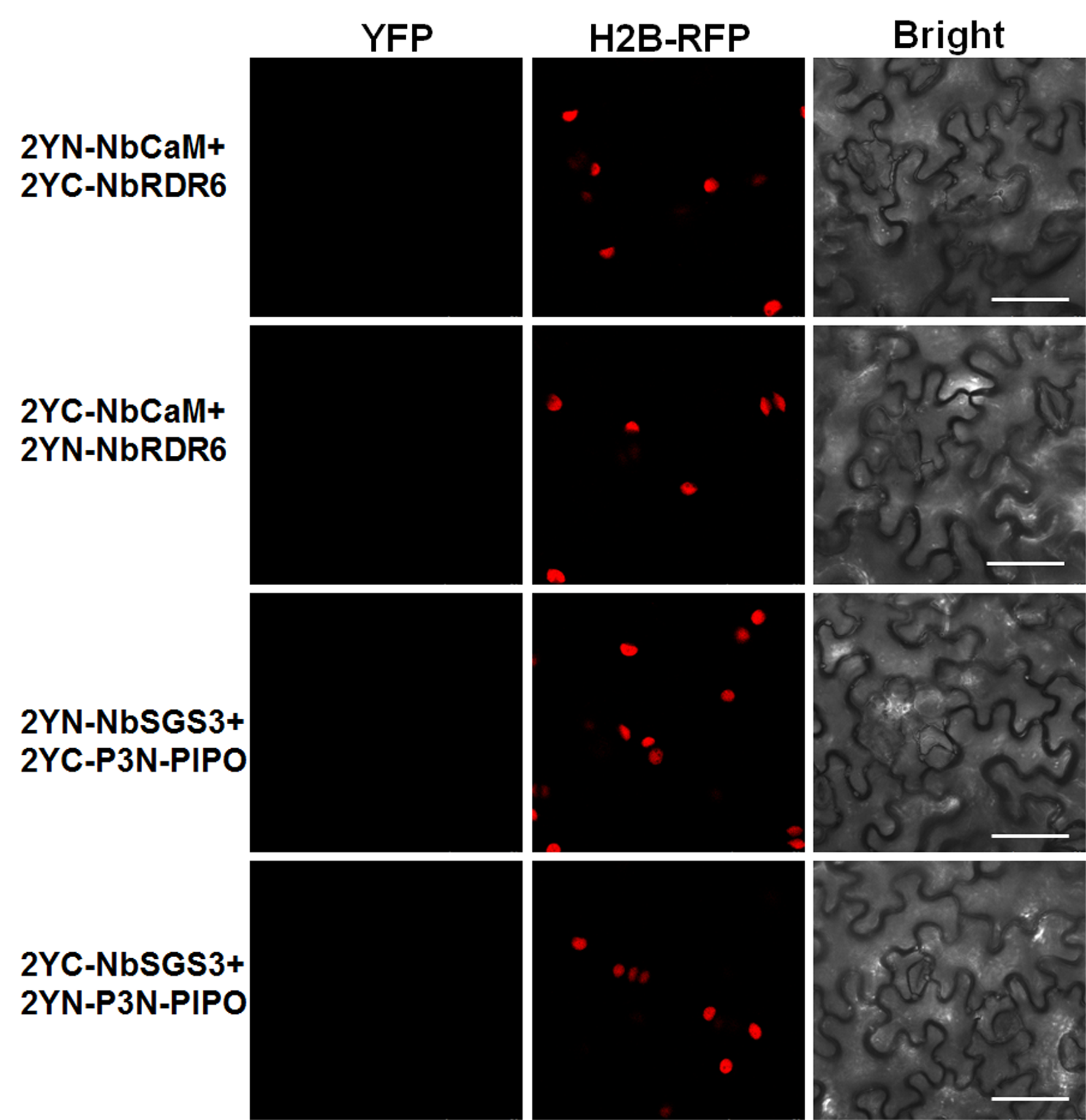

Supplement: S2 Fig — BiFC assays in N. benthamiana leaves expressing NbCaM and NbRDR6, or NbSGS3 and P3N-PIPO in H2B-RFP transgenic N. benthamiana leaves at 48 hours post infiltration (hpi). Bars = 50 μm. No YFP fluorescence was detected. (TIF) [file ppat.1006213.s003.tif]

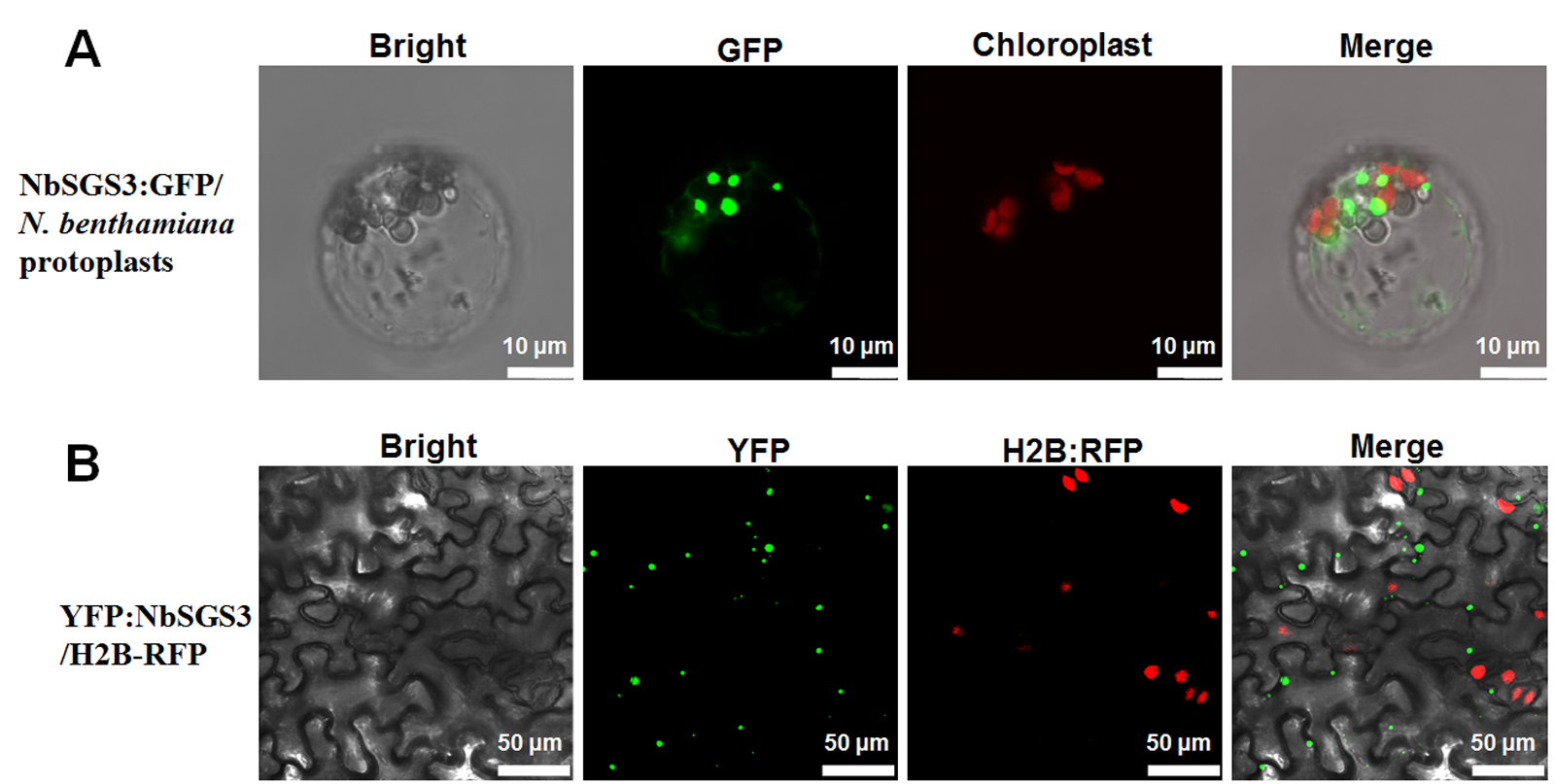

Supplement: S3 Fig — (A) Micrographs showing cells from protoplasts, which were prepared from N. benthamiana leaves transformed with 20 μg of 35S:NbSGS3:GFP plasmid DNA using polyethylene glycol (PEG)-mediated transformation, and micrographs taken 36 h after transformation. Bars = 10 μm. GFP fluorescence was detected in punctate spots in the cytoplasm. (B) Micrographs showing cells from leaves of H2B-RFP transgenic N. benthamiana expressing YFP:NbSGS3. Yellow fluorescence was detected from YFP:NbSGS3 (Green). Red fluorescence showed H2B-RFP as a nuclear marker. Bars = 50 μm. (TIF) [file ppat.1006213.s004.tif]

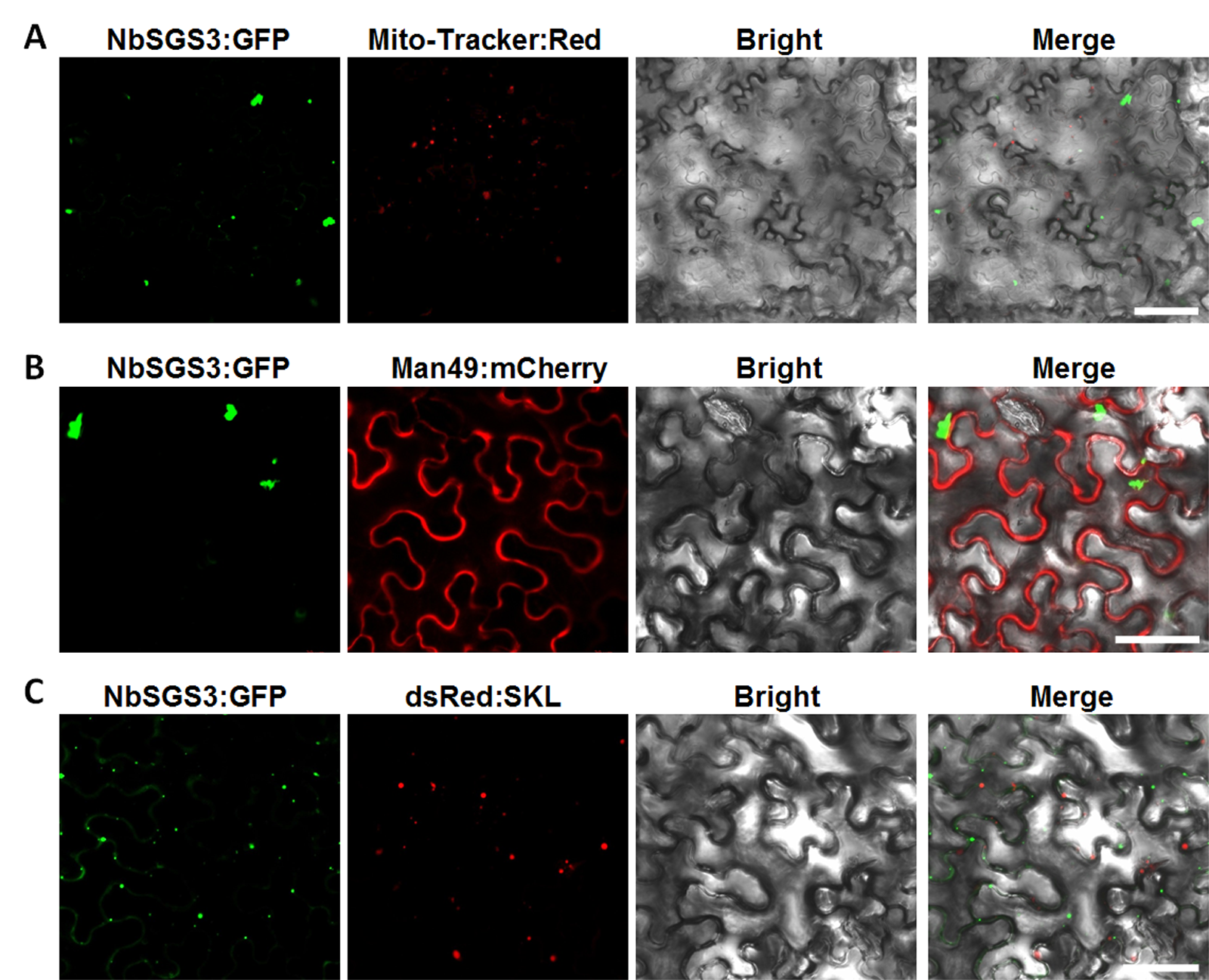

Supplement: S4 Fig — (A) Micrographs showing cells from N. benthamiana leaves firstly infiltrated with NbSGS3:GFP and then stained with mitochondria tracker red (Mito-Tracker:Red) at 48 hpi. (B) Micrographs showing cells co-expressing NbSGS3:GFP and golgi marker Man49:mCherry. (C) Micrographs showing cells co-expressing NbSGS3:GFP and peroxisomes marker dsRED:SKL. Bars = 50 μm. (TIF) [file ppat.1006213.s005.tif]

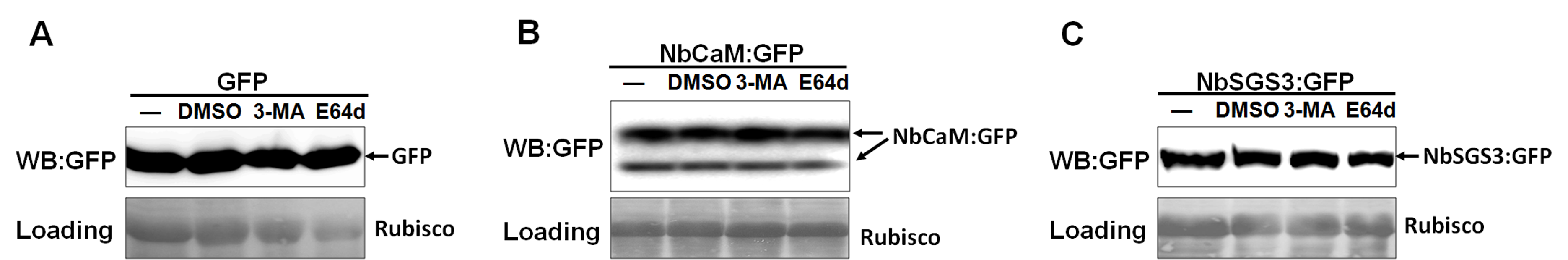

Supplement: S5 Fig — The effects of autophagy inhibitors 3-MA or E64d on the accumulation of GFP (A), NbCaM:GFP (B) or NbSGS3:GFP (C) protein detected by Western blot using GFP antibody. No treatment (-), DMSO, 3-MA (10 mM) or E64d (100 uM) treated samples were harvested from plants agroinfiltrated with GFP, NbCaM:GFP or NbSGS3:GFP. Ponceau staining of Rubisco large subunit was used as a loading control. (TIF) [file ppat.1006213.s006.tif]

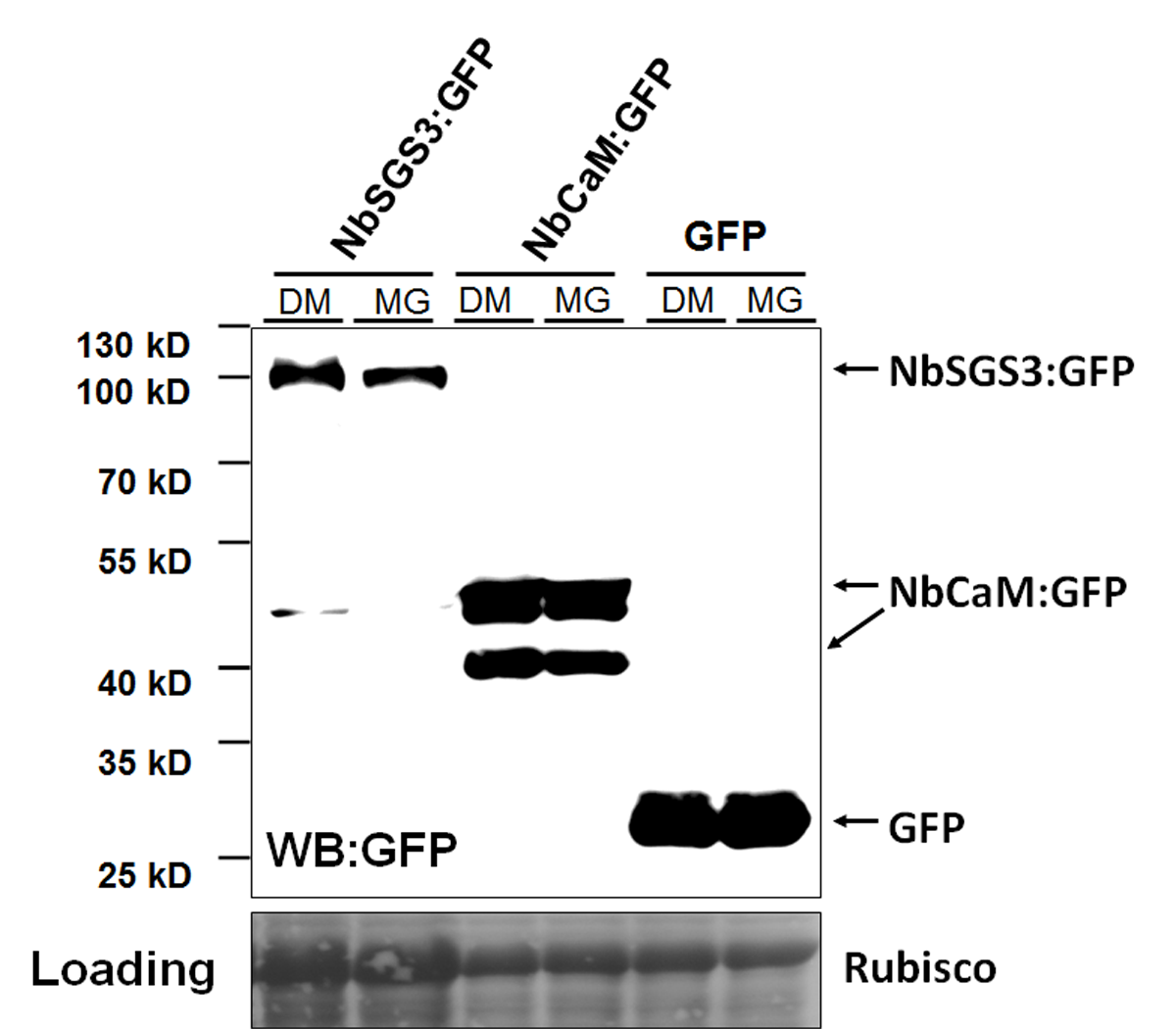

Supplement: S6 Fig — The effects of the 26S-proteasome inhibitor MG132 on accumulation of NbSGS3:GFP, NbCaM:GFP or GFP proteins were measured by WB using a GFP antibody (WB:GFP). DMSO (DM), or an equal volume of MG132 (100 μM) was infiltrated 16 h before the samples were harvested. Ponceau staining was used as a loading control. (TIF) [file ppat.1006213.s007.tif]

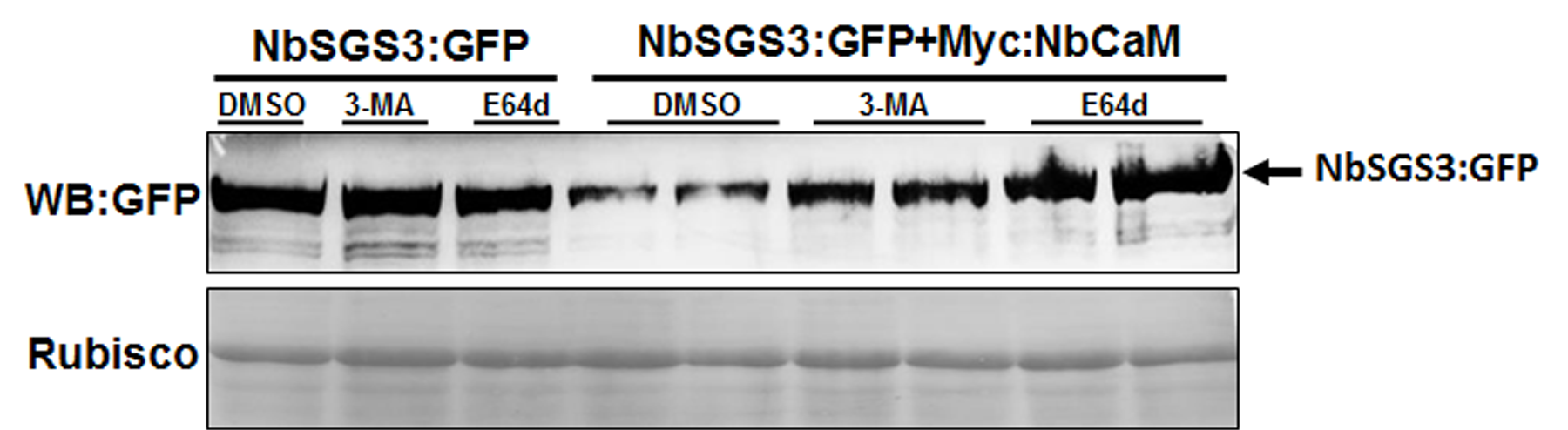

Supplement: S7 Fig — Samples were analyzed by Western blot using GFP antibody. Agrobacterium culture mixtures containing a plasmid capable of expressing NbSGS3:GFP alone, or together with Myc:NbCaM are indicated. DMSO (control), 3-MA (10 mM) or E64d (100 uM) was infiltrated into Nicotiana benthamiana leaves 32 h after being agroinfiltrated with NbSGS3:GFP alone or together with Myc:NbCaM constructs. Samples were harvested from 16 h later. Ponceau staining of Rubisco large subunit was used as a loading control. (TIF) [file ppat.1006213.s008.tif]

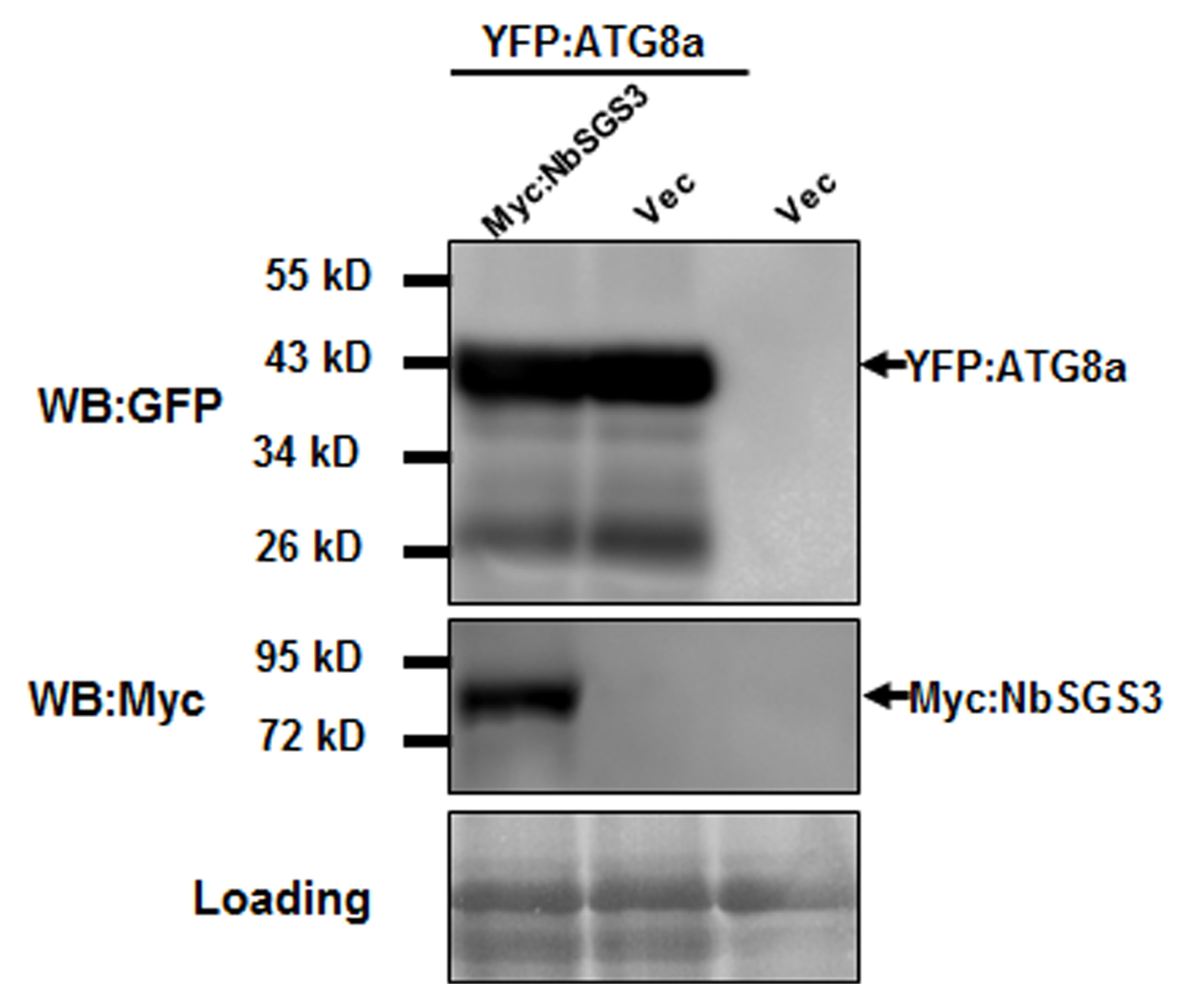

Supplement: S8 Fig — Samples were harvested from plants agroinfiltrated with YFP:ATG8a and Myc:NbSGS3 or empty vector (Vec) at 48 hours post infiltration. GFP (WB:GFP) and Myc (WB:Myc) antibodies were used in Western blot analysis and Coomassie brilliant blue staining of Rubisco large subunit was used as a loading control. (TIF) [file ppat.1006213.s009.tif]

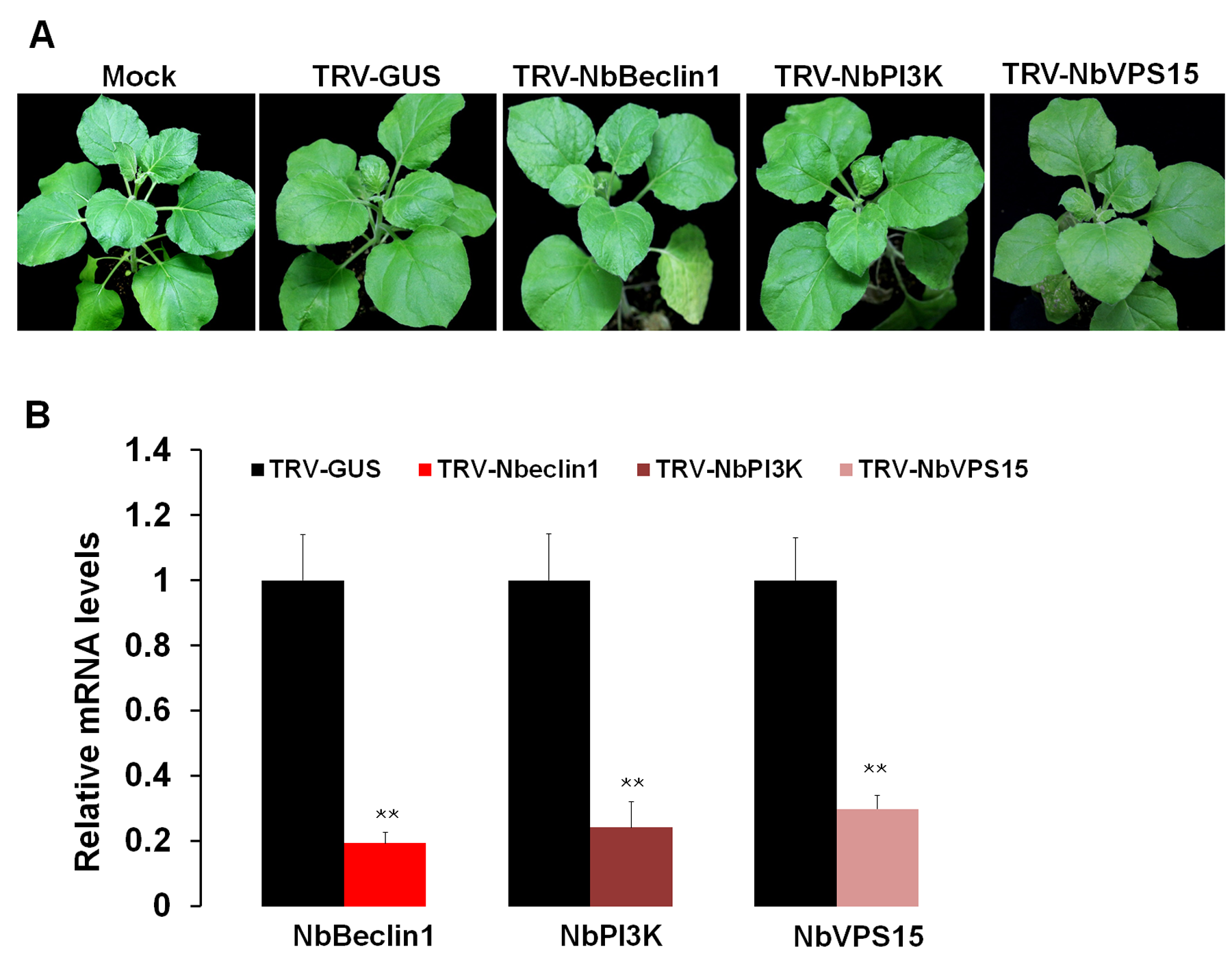

Supplement: S9 Fig — (A) The phenotype in mock, TRV-GUS, TRV-NbBeclin1, TRV-NbPI3K, or TRV-NbVPS15-treated plants at 21 dpi. Partial fragments of NbBeclin1, NbPI3K and NbVPS15 were cloned into RNA2 of the TRV VIGS vector. N. benthamiana plants at the 4–5 leaf stage were infiltrated with Agrobacterium cultures carrying an empty vector (mock), pTRV1 and pTRV2-GUS, or pTRV1 and pTRV2-VIGS. (B) Silencing of the indicated ATG genes (NbBeclin1, NbPI3K or NbVPS15) was confirmed in newly emerged leaves 14 dpi by RT-qPCR. Values for the ATG genes in TRV-GUS infected plants were arbitrarily set 1. Double asterisks indicate a highly significant difference (p<0.01) between TRV-GUS infected plants and TRV-ATGs infected plants (student’s t test). (TIF) [file ppat.1006213.s010.tif]

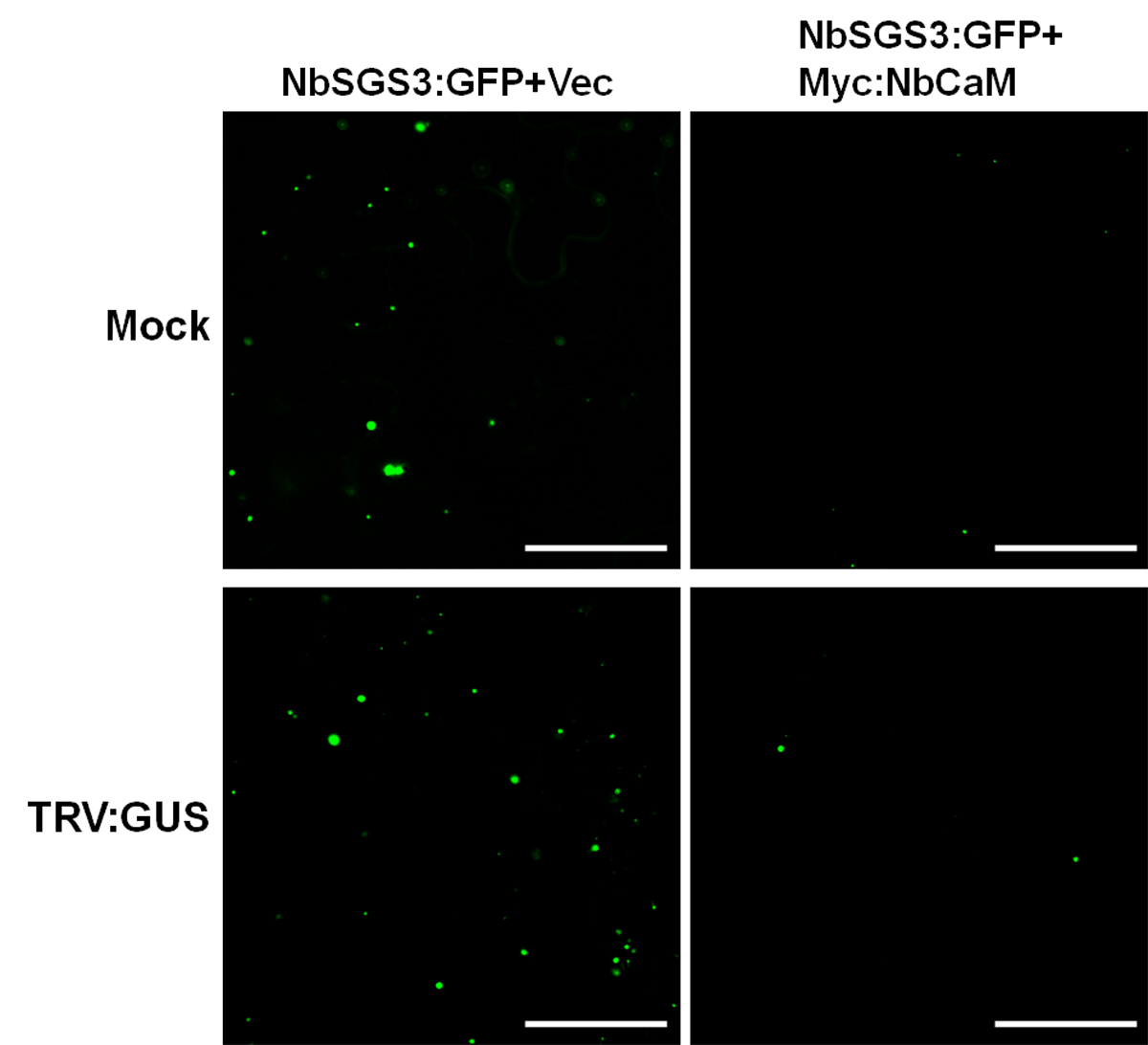

Supplement: S10 Fig — The newly leaves of mock (no TRV infection), or TRV-GUS-treated N. benthamiana plants were infiltrated with NbSGS3:GFP and empty vector (Vec) or NbSGS3:GFP and Myc:NbCaM at 21 dpi. Infiltrated leaves were examined at 48 hpi. Bars represent 50 μm. (TIF) [file ppat.1006213.s011.tif]

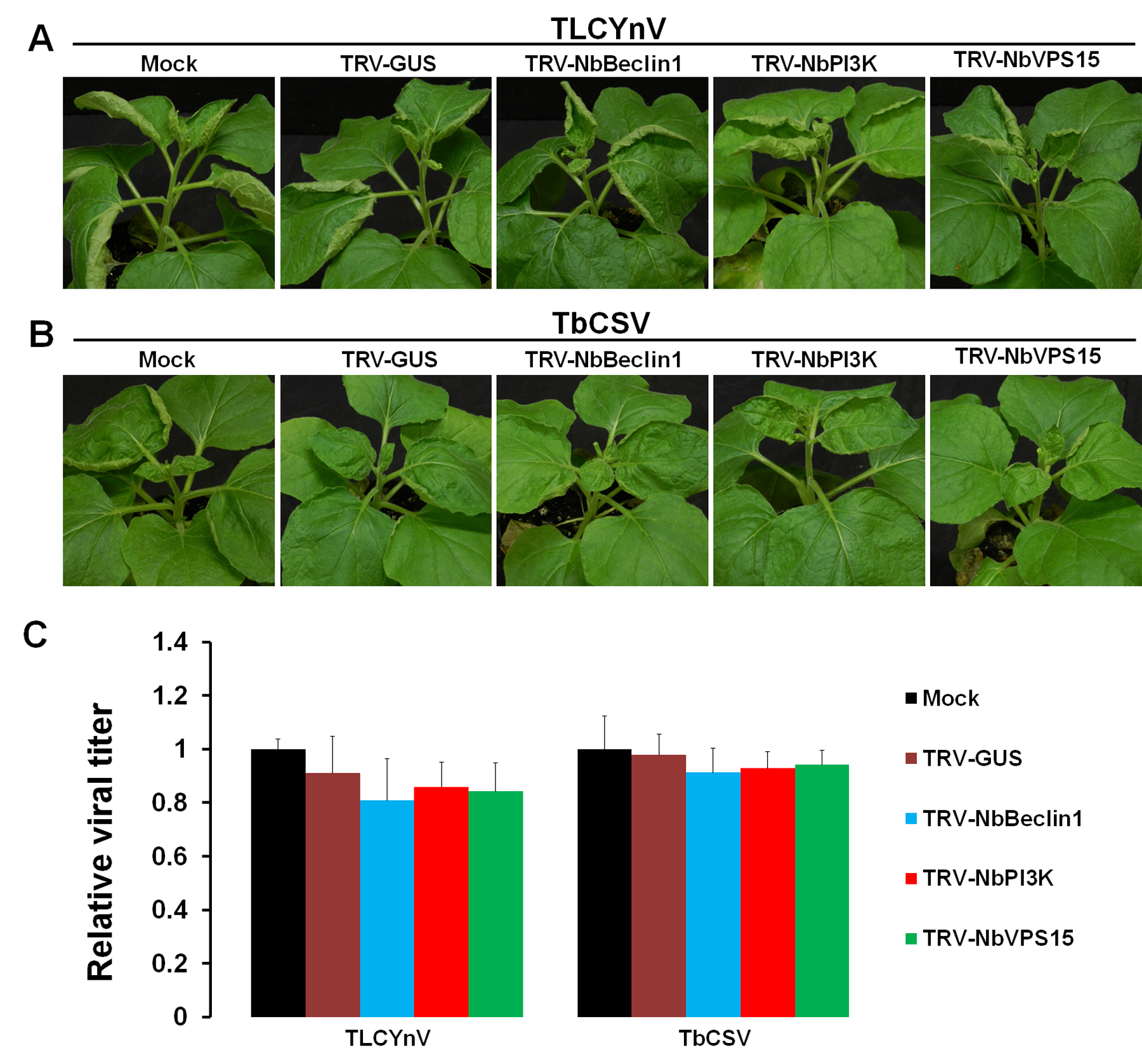

Supplement: S11 Fig — (A, B) Symptoms of mock, TRV-GUS and NbBeclin1, NbPI3K or NbVPS15-silenced N. benthamiana plants infected by TLCYnV (A) or TbCSV (B) at 14 dpi. (C) Relative TLCYnV or TbCSV DNA accumulation levels in plants shown in (A, B) normalized to 25S rRNA that served as an internal plant genomic DNA control. The upper newly infected leaves were harvested and 100 ng total DNA were used for relative quantitative PCR. The level of TLCYnV or TbCSV DNA in mock plants is arbitrarily set as 1. Values represent the mean ± standard deviation (SD) (n = 9). (TIF) [file ppat.1006213.s012.tif]

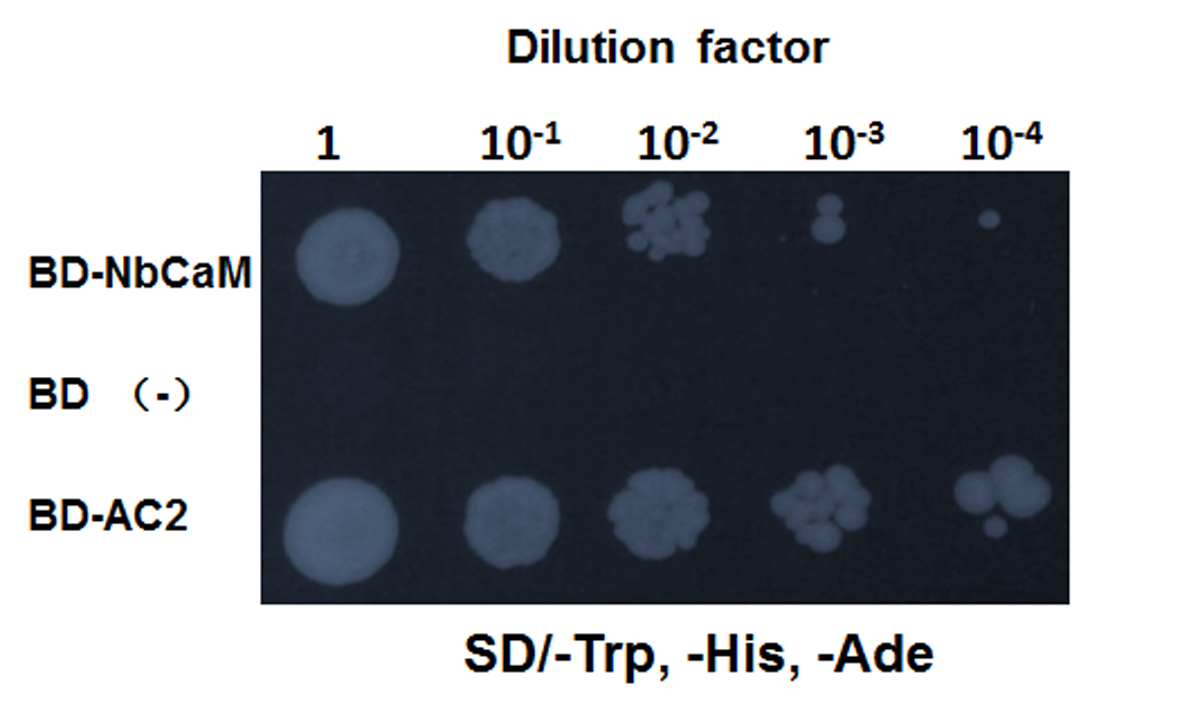

Supplement: S12 Fig — Yeast cells carrying AD+BD:NbCaM, BD alone (negative control) or AD+BD-AC2 (positive control) were cultured on the selective synthetic complete medium as indicated and then were photographed after 3 days. AC2 was cloned from Mungbean yellow mosaic virus. (TIF) [file ppat.1006213.s013.tif]
